# Diffusion distribution model for damage mitigation in scanning transmission electron microscopy

**DOI:** 10.1111/jmi.13351

**Published:** 2024-08-21

**Authors:** Amirafshar Moshtaghpour, Abner Velazco‐Torrejon, Daniel Nicholls, Alex W. Robinson, Angus I. Kirkland, Nigel D. Browning

**Affiliations:** ^1^ Correlated Imaging Theme, Rosalind Franklin Institute Harwell Science & Innovation Campus Didcot UK; ^2^ Department of Mechanical, Materials, & Aerospace Engineering University of Liverpool Liverpool UK; ^3^ Department of Materials University of Oxford Oxford UK

**Keywords:** beam damage, compressive sensing, diffusion distribution, scanning transmission electron microscopy

## Abstract

Despite the widespread use of Scanning Transmission Electron Microscopy (STEM) for observing the structure of materials at the atomic scale, a detailed understanding of some relevant electron beam damage mechanisms is limited. Recent reports suggest that certain types of damage can be modelled as a diffusion process and that the accumulation effects of this process must be kept low in order to reduce damage. We therefore develop an explicit mathematical formulation of spatiotemporal diffusion processes in STEM that take into account both instrument and sample parameters. Furthermore, our framework can aid the design of Diffusion Controlled Sampling (DCS) strategies using optimally selected probe positions in STEM, that constrain the cumulative diffusion distribution. Numerical simulations highlight the variability of the cumulative diffusion distribution for different experimental STEM configurations. These analytical and numerical frameworks can subsequently be used for careful design of 2‐ and 4‐dimensional STEM experiments where beam damage is minimised.

## 1 INTRODUCTION

(Scanning) Transmission Electron Microscopy (S(TEM)) is a widely used tool for investigating complex structures at the atomic level.^1^, ^2^, ^3^ This technique is now routine due to the development and implementation of spherical aberration correctors,^4^, ^5^, ^6^ improved electron sources^7^, ^8^, ^9^ and direct electron detectors.^10^, ^11^ For STEM imaging, a high intensity coherent, convergent probe is scanned over a region of interest of a sample,^12^, ^13^ which can lead to detrimental *beam damage*.^14^, ^15^

Beam damage can arise through various mechanisms. *Knock‐on* damage results from an electron‐atom interaction, whereby the incident electron transfers kinetic energy to an atom in the sample.^14^, ^16^ In this case, kinetic energy and momentum are conserved in the collision and an atom may be displaced from its equilibrium position or from its atomic site, if the transferred energy is higher than an atomic displacement energy.^17^

**FIGURE 1:**
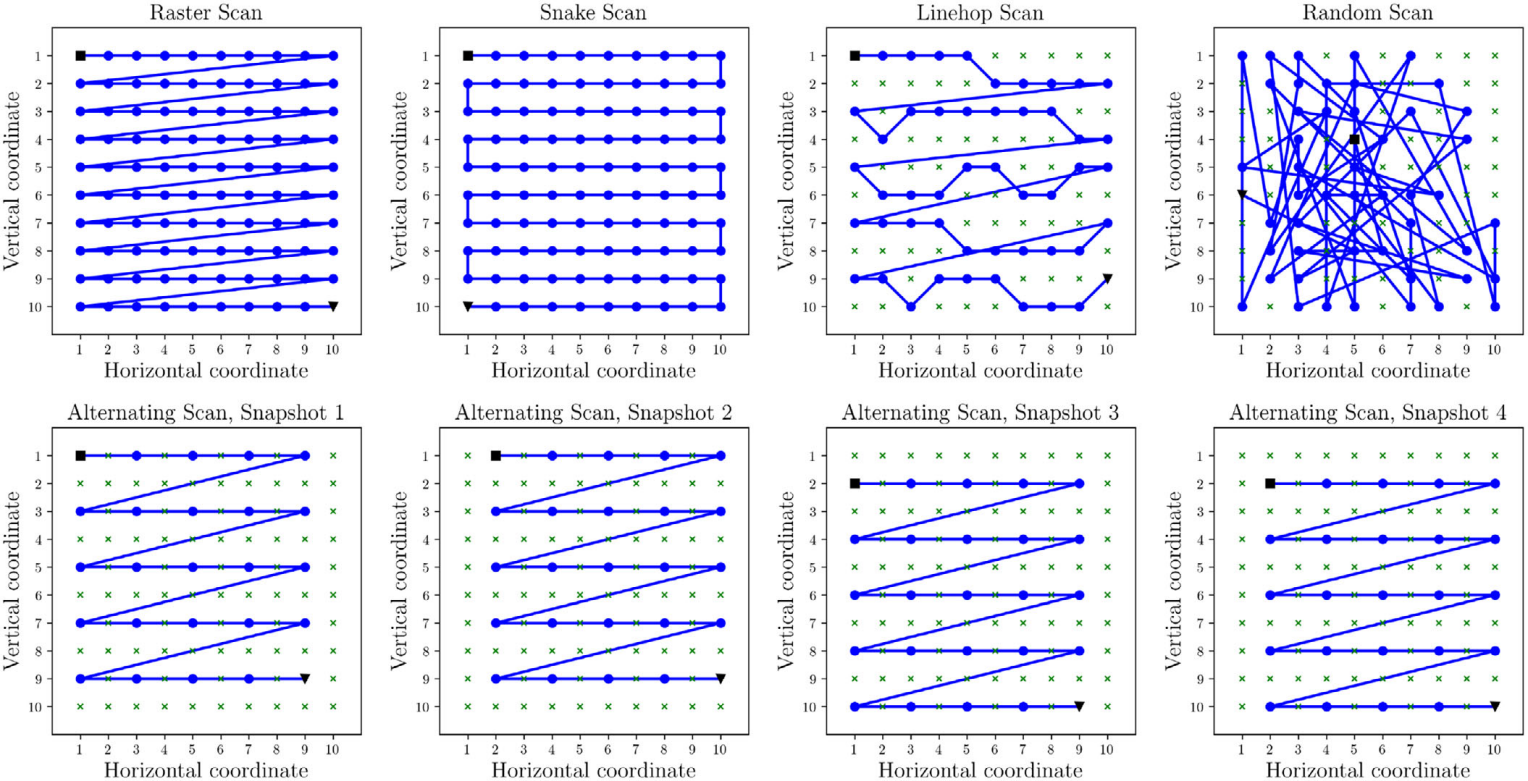
Scanning probe trajectories on a 10×10 grid. The first and last scanned probe positions are indicated by a black square and a triangle, respectively whereas the unscanned positions are shown by green crosses. Linehop and random scans are illustrated for 50% sampling of probe positions. Four snapshots associated with an alternating scan of order 2 are shown in the bottom panel. As for raster and snake scans, the alternating approach scans the full FoV.

A second mechanism, predominately affecting insulators, known as *radiolysis* describes the cleavage of chemical bonds within the structure of a sample.^17^ This occurs due to an inelastic electron‐electron interaction, whereby the incident electron causes either excitation or ionisation. If sufficient kinetic energy is transferred to a valence electron or an inner‐shell electron, this can cause the generation of secondary electrons, unstable radicals or ions that subsequently result in the dissociation of chemical bonds and eventually induce defect formation or even amorphisation. If the time for electron‐hole pair recombination is longer than a critical value, ions can migrate due to the Coulomb potential, which can be induced by the incident electron beam.^15^, ^17^ The secondary electrons that are released during this interaction can travel distances of the order of tens of nanometres, creating further damage away from the irradiated position.^18^, ^19^ These adverse effects can in principle be considered to be proportional to the electron fluence – that is, the number of electrons delivered over a region of interest. However, due to the delocalised effect of radiolysis, regions of the sample that are visited by the STEM probe at a later stage in the scanning sequence can be damaged when scanning earlier positions. In addition, because of the dynamic nature of radiolysis, due to the primary and secondary processes occurring on different timescales,^20^ conventional raster scan as shown in Figure 1 can induce more damage if the scan step and dwell time favour a fast accumulation of the damage effects. However, although the physical process(es), for example, electrostatic charging, diffusion of radicals, ions, and/or heat, affecting a particular sample^14^ may not be clearly identified, these mechanisms all behave as a diffusion process that extends spatially in time.

Emerging approaches for reducing beam damage are based on controlling the probe trajectory for scanning the full Field of View (FoV). A recent report^21^ has shown damage reduction using an alternating scan (also known as interleaved or leapfrog scan) compared to raster scan for high‐resolution imaging of a zeolite sample. As shown in Figure 1, in an alternating scan, all probe positions in a 2‐D array of points are visited by skipping a fixed number of points in both horizontal and vertical directions on the grid. A previous report^22^ exploited a random scan strategy, selecting the next probe location at random for hyperspectral STEM imaging to reduce emission instabilities in a cathodoluminescence experiment. In both examples, the same number of electrons are distributed over the scanned area as in the raster scan but adjacent probe positions are not used sequentially.

An alternative approach to mitigate beam damage is based on subsampling the probe positions. A high‐quality reconstruction is expected assuming that the image has an (approximately) sparse representation in a transform domain.^23^ Subsampled STEM modalities are often recognised as applications of the theory of Compressed Sensing (CS)^23^, ^24^ in the field of electron microscopy, subsequently, referred to as *compressive STEM* for convenience. This subsampled acquisition allows for a larger average distance between neighbouring positions compared to a conventional full acquisition. Experimentally, subsampling of the probe positions can be achieved using a scan generator or a beam blanker.^25^, ^26^ A scan generator is used to alter the trajectory of the probe such that only the desired positions within the FoV are sampled. A beam blanker, without changing the trajectory of the probe from a default raster scan, deflects the beam rapidly and selects locations that are irradiated along the scan path.

Other approaches such as the fractionation of the fluence by multiframe fast acquisitions have also shown damage reduction in contrast to a single acquisition, for a fixed total fluence in STEM spectrum imaging of a lead perovskite.^27^ Importantly, these findings support the diffusion‐like behaviour of radiolysis and suggest that some of the damage processes of this mechanism are reversible.

As described earlier, the displacement of atoms by *knock‐on* involves a simplistic mechanism, and a relatively large number of analytical and molecular dynamic models exist to describe and model that interaction.^28^ However, because of the complexity of the multiple processes triggered by radiolysis, there is a lack of models that fully describe this mechanism, which has limited the progress of experimental strategies to mitigate its effects. A diffusion process, supported by experimental results, is therefore an attractive concept that can be implemented as an analytical Fick's diffusion model.^29^, ^30^

### 1.1 Background and outline

Previous studies have used diffusion models to model electron beam damage and its propagation through a sample during a STEM acquisition process.^31^, ^32^, ^33^ Nicholls et al.^32^ first used this approach to provide insights as to why subsampled measurements (as in compressive STEM) reduce beam damage. That work also demonstrated that linehop sampling,^25^ a sampling method designed to overcome scan coil hysteresis, is as effective as random sampling for reducing beam damage at low sampling rates (6.25% of scanning probe positions). As shown in Figure 1, linehop sampling involves constructing an image from a series of lanes, where each lane only samples one pixel from each column, and where the next pixel to be sampled is a neighbour to the previous column. This limits the range of motion during sampling while still allowing random perturbations, satisfying both the conditions for hysteresis from the scan coils and incoherence for data recovery using inpainting.^23^

Jannis et al.^33^ have extended that work to explore experimental results obtained from alternating scan strategies in STEM. In this work, a 2‐D diffusion process from a continuous point source is considered as the mediator of damage, and it was shown that by the implementation of a damage threshold the proposed model could explain the experimental results for a reduction in damage using this scan strategy compared to a raster scan. Since the detailed physics of the damage process may not be known, the concentration of a diffusing substance in the model is replaced by a parameter defined as a ‘state of the sample’, which is altered by the electron beam. Sample damage then only occurs when that parameter crosses a defined threshold. The total damage at a certain position is thus calculated by integrating a nonlinear function, referred in this paper to as a damage activation function, over time.

The focus of this work is to further develop existing models^32^, ^33^ and to produce a rigorous mathematical framework for these. Following the work by Jannis et al.,^33^ we make use of a damage threshold and include a damage diffusion distribution, or diffusion distribution for short, to replace the classical concentration of a diffusing substance. We emphasise that beam damage always occurs and the detection of the related processes is limited by the sensitivity of the detection systems. The threshold is used in our mathematical approach to limit the accumulation of the ‘diffusion distribution’ and was previously proposed from observations of damage reduction in STEM experiments using different scan geometries.^34^

This work provides the first explicit formulations of a damage induced diffusion process model in STEM by considering a realistic nonpoint source representation of the electron probe. We note that since our mathematical model supports an electron probe of arbitrary size, the findings of this work can be directly applied to 4‐D STEM scans ^35^, ^36^, ^37^. This enables future studies of diffusion distributions (i) in three dimensions, (ii) from pulsed (or modulated) electron beams, or (iii) in a TEM geometry. A summary of the key findings reported in this paper are:

- Based on the Fick's second model,^29^ we derive the spatiotemporal diffusion distribution of an instantaneous point source in a d‐dimensional medium with an anisotropic diffusion coefficient (Equation 2). This is a generalisation of the previous formulation in Ref. [33] from a two‐ to d‐dimensional medium and from isotropic to anisotropic diffusion coefficients.
- Using that solution and the principle of superposition, we provide the diffusion distribution for an arbitrary‐shaped source (see Equation 3).
- We extend that model to a continuous source and provide closed‐form formulations for diffusion distributions for four special cases of continuous sources: (i) point source, (ii) square disc source, (iii) circular disc source and (iv) Gaussian‐shaped source.
- Our analyses of diffusion distribution in STEM assume that the 2‐D Gaussian function is a close proxy to a more realistic airy disc ^2^ function. By computing the first and second derivatives of the diffusion distribution with respect to both space and time, we investigate the spatiotemporal behaviour of the diffusion distribution. Our findings in Lemmas S5, S6, S11, and S12 highlight the complexity of the diffusion process caused by activating an electron probe at a single location.
- We formulate in Section 3.2 a Cumulative Diffusion Distribution (CDD) caused by activating an electron probe at multiple locations during STEM acquisitions. The CDD takes into account the diffusion distributions caused not only by the currently activated electron probe but also by every electron probe activated previously.
- Our STEM diffusion model can be coupled to a damage threshold mechanism (Equation 23). We propose two Diffusion Induced Damage (DID) parameters: (i) the frequency of DID events, and (ii) the intensity of DID events. For each of these, we distinguish online vs. offline damage observations. In the online case, the DID at a certain location is affected only until that location is scanned, whereas in the offline case, DID changes at every location – regardless of whether it is scanned later on or not. Moreover, in Theorem 2, we provide sufficient mathematical conditions, for which subsampling probe positions, as in compressive STEM, reduces the DID compared to full sampling. That result supports the experimental findings in the literature reporting the advantageous of subsampling probe positions^32^, ^33^, ^38^, ^39^ in reducing damage.
- We present extensive numerical simulations, in Section 6, which illustrate the advantages of the proposed framework.
- Finally, in Sections 5.1 and 6.5, for given STEM acquisition parameters, we have designed a Diffusion Controlled Sampling (DCS) strategy that selects as many probe positions as possible while ensuring that the global maximum of the CDD is constrained, or equivalently the DID is minimised.

## 2 A MATHEMATICAL MODEL FOR DIFFUSION

We base our analysis of diffusion distribution on the second Fick's model [30, §1.2], in which the diffusion distribution (also known as the diffusion concentration) ϕ of some diffusing species in a medium at a d‐dimensional spatial location^1^ $r={\left[r_{1},\text{…},r_{d}\right]}^{⊤}\in R^{d}$ and at time t>0 is the solution of the following Partial Differential Equation (PDE) [Ref. 30, equation 1.5], that is,

(1) $$\frac{\partial \phi (r,t)}{\partial t}=\sum_{l=1}^{d}D_{l}\frac{\partial^{2}\phi \left(r,t\right)}{\partial r_{l}^{2}}.$$

where $D_{l}>0$, in $m^{2}\cdot s^{-1}$, is the diffusion coefficient along the lth coordinate. Equation (1) assumes that the medium is homogeneous such that the diffusion coefficient does not vary from point to point. It also assumes that the diffusion coefficient is independent of the concentration of a diffusing substance ϕ as in, for example the diffusion of organic vapours in high‐polymer substances. In the remaining of this paper, we assume that this diffusion is caused by an electron source interacting with an infinite medium; hence, no boundary condition is required to solve Equation (1).

For an instantaneous point electron source activated at a location $r=r_{0}$ and time $t=t_{0}$, the solution of the PDE in Equation (1) is (Section S1 gives the derivation)

(2) $$\phi \left(r,t\right)=\frac{Q_{0}}{\sqrt{\left|4\pi D\right|{\left(t-t_{0}\right)}^{d}}}\exp\left(-\frac{{\left(r-r_{0}\right)}^{⊤}D^{-1}\left(r-r_{0}\right)}{4(t-t_{0})}\right),$$

where $Q_{0}$ is the initial rate of the diffusing species, and the diagonal matrix $D≔\mathrm{diag}\left(D_{1},\text{…},D_{d}\right)\in R^{d\times d}$ contains the diffusion coefficients on its diagonal with |D| denoting the determinant of D. Equation (2) extends previous results (Ref. 30, equation 2.6, Ref. 40, p. 150, and Ref. 41) to a d‐dimensional anisotropic diffusion model, which is equivalent to nonidentical diffusion coefficients along different coordinates.

Remark 1 In Equation (2) and subsequently, we assume that $Q_{0}$ is related to the electron beam current $I_{0}$ through a general function $M:R_{\geq 0}\mapsto R_{\geq 0}$, that is, $Q_{0}=M\left(I_{0}\right)$. For the sake of convenience, we further assume the units of the rate of the diffusing species $Q_{0}$ to be $u\cdot s^{-1}$ for an unspecified and arbitrary ‘u’ here; hence, the unit of the diffusion distribution ϕ is $u\cdot m^{-2}$. This assumption allows a comparison of different STEM scans with fixed beam current. By comparison the authors in Ref. [33] considered that the electron probe deposits energy at every probe location and assumed $Q_{0}$ with units of $s^{-1}$. However, we emphasise that we do not make any claim about the nature of the diffusive species in STEM.

Using the principle of superposition [Ref. 30, equation 3.5a], the solution of the PDE in (1) for a source with an arbitrary spatiotemporal activation profile h(r,t) can be written as:

(3) $$\begin{array}{ccc} \phi (r,t) & = & \int_{0}^{t}\int_{R^{d}}h\left(r^{\prime },t^{\prime }\right)\frac{1}{\sqrt{\left|4\pi D\right|{\left(t-t^{\prime }\right)}^{d}}}\\ & & \exp\left(-\frac{{\left(r-r^{\prime }\right)}^{⊤}D^{-1}\left(r-r^{\prime }\right)}{4(t-t^{\prime })}\right)dr^{\prime }dt^{\prime }. \end{array}$$

It is straightforward to confirm that the total number of diffusing species $Q_{\mathrm{tot}}\left(t\right)$ at time t in a system with a diffusion distribution given by Equation (3) is equal to the total number of units deposited by the source from the activation time of the source to the current time, since

(4) $$Q_{\mathrm{tot}}\left(t\right)≔\int_{R^{d}}\phi \left(r,t\right)\;dr=\int_{0}^{t}\int_{R^{d}}h\left(r^{\prime },t^{\prime }\right)\;dr^{\prime }dt^{\prime }.$$

### 2.1 Special cases in 2‐D

From Equation (3), we now develop the diffusion distribution formulation for specific cases of continuous sources in a 2‐D medium. From 1 we recall that the units of diffusion distribution in 2‐D are $u\cdot {\mathrm{nm}}^{-2}$.

#### Continuous point source activated at location $r=r_{0}$ during time $t\in [t_{0},t_{0}+\tau ]$:

In this case, the corresponding source function can be decomposed into spatial and temporal components, that is, $h\left(r,t\right)=Q_{0}\cdot h_{s}\left(r\right)\cdot h_{t}\left(t\right)$ with

(5) $$h_{s}\left(r\right)=\delta_{r_{0}}\left(r\right)\;\mathrm{and}\;h_{t}\left(t\right)=\left\{\begin{array}{cc} 1, & \mathrm{if}\;t_{0}\leq t\leq t_{0}+\tau ,\\ 0, & \mathrm{otherwise}, \end{array}\right.$$

where $Q_{0}$, in $u\cdot s^{-1}$ and $\delta_{r_{0}}\left(r\right)=+\infty$, if $r=r_{0}$, and $\delta_{r_{0}}\left(r\right)=0$, if $r\neq r_{0}$, is the delta Dirac function, whose integral over the entire domain is unity: $\int_{R^{d}}\delta_{r_{0}}\left(r\right)dr=1$. Inserting Equation (5) in Equation (3) for d=2 dimensions gives (Section S2 gives details);

(6) $$\begin{array}{ccc} & & \phi (r,t)=\\ & & \left\{\begin{array}{cc} \frac{Q_{0}}{4\pi \sqrt{\left|D\right|}}E_{1}\left(\frac{{\left(r\;-\;r_{0}\right)}^{⊤}D^{-1}\left(r\;-\;r_{0}\right)}{4(t-t_{0})}\right), & t_{0}\leq t\leq t_{0}+\tau ,\\ \frac{Q_{0}}{4\pi \sqrt{\left|D\right|}}\left(E_{1}\left(\frac{{\left(r\;-\;r_{0}\right)}^{⊤}D^{-1}\left(r\;-\;r_{0}\right)}{4(t-t_{0})}\right)\right. & \\ -\left.E_{1}\left(\frac{{\left(r\;-\;r_{0}\right)}^{⊤}D^{-1}\left(r\;-\;r_{0}\right)}{4(t-\left(t_{0}+\tau \right))}\right)\right), & t>t_{0}+\tau , \end{array}\right. \end{array}$$

at any spatial point other than the activation point $r\neq r_{0}$, and at the activation point $r=r_{0}$,

$$\phi \left(r_{0},t\right)=\left\{\begin{array}{cc} +\infty , & t_{0}\leq t\leq t_{0}+\tau ,\\ \frac{Q_{0}}{4\pi \sqrt{\left|D\right|}}\ln\left(\frac{t-t_{0}}{t-(t_{0}+\tau )}\right), & t>t_{0}+\tau . \end{array}\right.$$

**FIGURE 2:**
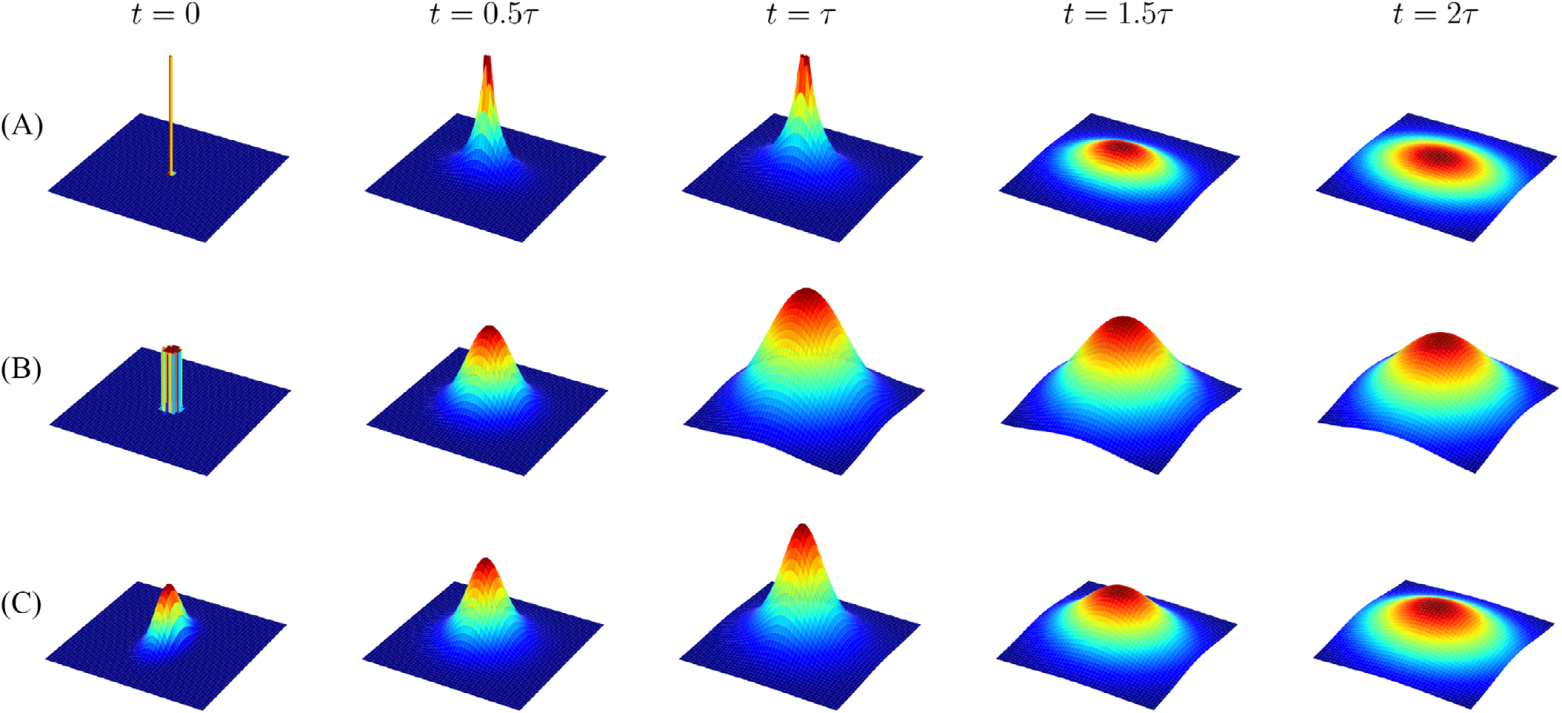
Diffusion distributions for three special cases in Section 2. (A) Point source: Equation (6) with D=diag(0.25,0.5). (B) Circular disc source: Equation 8 with D=0.25 and $r_{s}=0.2$. (C) Gaussian‐shape source: Equation 10 with $D_{s}=\mathrm{diag}\left(0.2,0.05\right)$ and D=diag(0.25,0.5). For all cases τ=1, $Q_{0}=1$, $t_{0}=0$, and $r_{0}=0$. The range of vertical axes are identical intra‐row but adjusted inter‐row for better visualisation.

In Equation (6), $E_{1}\left(v\right)≔\int_{v}^{+\infty }\frac{1}{u}e^{-u}\;du$ for v∈R/{0} and $E_{1}\left(0\right)=+\infty$ is the Exponential integral of order one. For an isotropic medium with a diffusion coefficient D or with a matrix of diffusion coefficients D=diag(D,D) Equation (6) reduces to Equation (1) in Ref. [33]. However, the value of the diffusion distribution at the activation point $\phi (r_{0},t)$ when t≥τ is not included in Ref. [33] as illustrated in Figure 2A.

This special case is relevant to imaging applications using STEM as studied in Ref. [33] (20). However, experimentally the source is not a point. Therefore, in the following we provide solutions to (1) for non‐point sources.

#### Continuous circular disc source with radius $r_{s}$ centered at $r=r_{0}$ and activated during time $t\in [t_{0},t_{0}+\tau ]$:

In this case, the corresponding source function is $h\left(r,t\right)=Q_{0}\cdot h_{s}\left(r\right)\cdot h_{t}\left(t\right)$ with

(7) $$h_{s}\left(r\right)=\left\{\begin{array}{cc} \frac{1}{\pi r_{s}^{2}}, & \mathrm{if}\;∥r-r_{0}{∥}_{2}\leq r_{s},\\ 0, & \mathrm{otherwise}, \end{array}\right.\;\mathrm{and}\;h_{t}\left(t\right)=\left\{\begin{array}{cc} 1, & \mathrm{if}\;t_{0}\leq t\leq t_{0}+\tau ,\\ 0, & \mathrm{otherwise}, \end{array}\right.$$

where the rate of charge $Q_{0}$ is $u\cdot s^{-1}$. In Equation (7), the $\ell_{2}$‐norm ${∥u∥}_{2}≔{\left(\sum_{i}|u_{i}{|}^{2}\right)}^{1/2}$ is the square root of the sum of the squared vector values and is used to model the circular disc source. For simplicity, we assume isotropic diffusion coefficients, that is, $D_{1}=D_{2}=D$. Inserting (7) in (3) for d=2 gives (see Section S3 for details)

(8) $$\begin{array}{ccc} \phi (r,t) & = & \int_{t_{0}}^{\min(t,t_{0}+\tau )}\frac{Q_{0}}{2\pi r_{s}^{2}D\left(t-t^{\prime }\right)}\exp\left(-\frac{∥r-r_{0}{∥}_{2}^{2}}{4D(t-t^{\prime })}\right)\\ & & \int_{0}^{r_{s}}u\exp\left(-\frac{u^{2}}{4D(t-t^{\prime })}\right)I_{0}\left(\frac{u∥r-r_{0}{∥}_{2}}{2D(t-t^{\prime })}\right)du\;dt^{\prime }, \end{array}$$

where $I_{0}\left(v\right)≔\frac{1}{\pi }\int_{0}^{\pi }\exp\left(v\cos\theta \right)d\theta$ is the modified Bessel function of the first kind of order zero.

This case is pertinent to imaging using wide (parallel) beams as in conventional TEM or Fourier Ptychography ^42^ and in certain X‐ray applications.^43^, ^44^ For completeness in S4, we extend our analysis to continuous square disc source.

**Continuous Gaussian source with a shape matrix** $D_{s}=\mathrm{diag}\left(D_{s,1},D_{s,2}\right)$ **centered at** $r=r_{0}$ **and activated during time** $t\in [t_{0},t_{0}+\tau ]$: in this case, the corresponding source function is $h\left(r,t\right)=Q_{0}\cdot h_{s}\left(r\right)\cdot h_{t}\left(t\right)$ with

(9) $$\begin{array}{ccc} h_{s}\left(r\right) & = & \frac{1}{\sqrt{|4\pi D_{s}|}}\exp\left(-\frac{1}{2}{\left(r-r_{0}\right)}^{⊤}D_{s}^{-1}\left(r-r_{0}\right)\right)\;\mathrm{and}\\ & & h_{t}\left(t\right)=\left\{\begin{array}{cc} 1, & \mathrm{if}\;t_{0}\leq t\leq t_{0}+\tau ,\\ 0, & \mathrm{otherwise}, \end{array}\right. \end{array}$$

where the rate of charge $Q_{0}$ is $u\cdot s^{-1}$. Inserting (9) in (3) for d=2 gives (see Section S5 for details)

(10) $$\begin{array}{ccc} \phi (r,t) & = & \int_{t_{0}}^{\min(t,t_{0}+\tau )}\frac{Q_{0}}{2\pi \sqrt{|D_{e}|}}\\ & & \exp\left(-\frac{1}{2}{\left(r-r_{0}\right)}^{⊤}D_{e}^{-1}\left(r-r_{0}\right)\right)\;dt^{\prime }, \end{array}$$

with $D_{e}=D_{s}+2\left(t-t^{\prime }\right)D$. Furthermore, assuming an isotropic source and diffusion coefficients, that is, $D_{s,1}=D_{s,2}=D_{s}$ and $D_{1}=D_{2}=D$, (10) reduces to

(11) $$\phi \left(r,t\right)=\left\{\begin{array}{cc} \frac{Q_{0}}{4\pi D}\left(E_{1}\left(\frac{∥r\;-\;r_{0}{∥}_{2}^{2}}{2D_{s}+4D\left(t-t_{0}\right)}\right)-E_{1}\left(\frac{∥r\;-\;r_{0}{∥}_{2}^{2}}{2D_{s}}\right)\right), & t_{0}\leq t\leq t_{0}+\tau ,\\ \frac{Q_{0}}{4\pi D}\left(E_{1}\left(\frac{∥r\;-\;r_{0}{∥}_{2}^{2}}{2D_{s}+4D\left(t-t_{0}\right)}\right)-E_{1}\left(\frac{∥r\;-\;r_{0}{∥}_{2}^{2}}{2D_{s}\;+\;4D\left(t-\left(t_{0}+\tau \right)\right)}\right)\right), & t>t_{0}+\tau , \end{array}\right.$$

for $r\neq r_{0}$ and

(12) $$\phi \left(r_{0},t\right)=\left\{\begin{array}{cc} \frac{Q_{0}}{4\pi D}\ln\left(\frac{D_{s}\;+\;2D\left(t\;-\;t_{0}\right)}{D_{s}}\right), & t_{0}\leq t\leq t_{0}+\tau ,\\ \frac{Q_{0}}{4\pi D}\ln\left(\frac{D_{s}\;+\;2D\left(t\;-\;t_{0}\right)}{D_{s}\;+\;2D\left(t\;-\;t_{0}-\tau \right)}\right), & t>t_{0}+\tau . \end{array}\right.$$

This special case, which forms the basis of the discussion in the remainder of this paper, is a good approximation for focused or defocused electron beams^45^ in the absence of any non‐circular aberrations.

Remark 2 By computing the integral in Equation (4) for the above source functions described by Equations (5), (7), and (9), the total number of diffusing species $Q_{\mathrm{tot}}\left(t\right)$ at time t in all systems is

$$Q_{\mathrm{tot}}\left(t\right)=Q_{0}\cdot \min\left(t\;-\;t_{0},\tau \right).$$

We note that the normalisation factors in the spatial component of the activation functions in Equations (7) and (9) mean that the number of diffusing species in the system is constant when the size of the source changes. Since by definition, the function M maps every value of the beam current to a single value of the diffusing species, this normalisation of the electron beam is consistent with the optical conditions available in modern STEM instruments: for a fixed beam current, changing the electron probe size does not affect the number of electrons (and in turn, the number of diffusing species) in the system.

## 3 DIFFUSION IN STEM

We start by introducing a general formulation of diffusion processes in STEM. Subsequently, we restrict our analysis to the following assumptions:

(A1) The rate of initial diffusing species $Q_{0}$ is related to the beam current through a general function M. It is not necessary to specify the function M and we here assume that the units of diffusion distribution is $u\cdot m^{-2}$ where ‘u’ is an arbitrary unit independent of the dwell time, diffusion coefficient, and probe size;
(A2) The sample is uniform and infinitesimally thin and is significantly larger than the probe;
(A3) The diffusion coefficient within the sample is constant;
(A4) An airy disk electron probe can be approximated by a Gaussian probe;
(A5) The number of diffusing species in the system is constant with respect to changes in the size of the electron probe;
(A6) The electron beam moves instantaneously and without errors between scan positions.

Assumption (A1) is a restatement of Remark 1. Assumption (A2) implies that diffusion in STEM can be mathematically simplified as a 2‐D process in an infinite medium. Accordingly, although beam damage occurs in three dimensions, we have simplified our study to two dimensions since the information obtained in a STEM image comes from a 2‐D projection of an object. Experimental parameters related to damage (such as the diffusion constant) estimated from STEM images, hence, only contain information about the process in the axial direction perpendicular to the sample. With a similar 2‐D model, Jannis et al.^33^ and Velazco et al.^34^ have shown qualitative correlation between experimental results and simulations. Assumptions (A3) and (A4) together allow the use of the diffusion profile of a Gaussian‐shaped source as formulated in Equation (11). Assumption (A5) is made for convenience to ensure that the beam current is constant with respect to changes in the probe conditions. Assumption (A6) neglects the real finite time response of the scan coils, which can introduce errors in the trajectory of the beam or its actual position. These can be illustrated by flyback, which is the trajectory of the beam between the end of a scan line and the beginning of the next one in a raster scan. This sudden jump of the beam creates errors in its trajectory that can be alleviated by applying a delay time at the start of each scan line.^46^

### 3.1 Diffusion distribution of an electron probe activated at a single location

In this section, we study the behaviour of the diffusion profile of a single electron probe (a probe activated at a single location). Following the assumptions above and from Equations (11) and (12), the diffusion distribution of the ith scanned electron probe positions with square width $D_{s}$, activated at location $r_{i}$ and time $t_{i}$, and during a dwell‐time $\tau_{i}$ is

(13) $$\phi_{i}^{\mathrm{on}}\left(r,t\right)=\left\{\begin{array}{cc} \frac{Q_{0}}{4\pi D}\left(E_{1}\left(\frac{∥r\;-\;r_{i}{∥}_{2}^{2}}{2D_{s}+4D\left(t-t_{i}\right)}\right)-E_{1}\left(\frac{∥r\;-\;r_{i}{∥}_{2}^{2}}{2D_{s}}\right)\right), & \mathrm{if}\;r\neq r_{i},\\ \frac{Q_{0}}{4\pi D}\ln\left(\frac{D_{s}\;+\;2D\left(t-t_{i}\right)}{D_{s}}\right), & \mathrm{if}\;r=r_{i}, \end{array}\right.$$

When the beam is on, that is, $t_{i}\leq t\leq t_{i}+\tau_{i}$, and

(14) $$\phi_{i}^{\mathrm{off}}\left(r,t\right)=\left\{\begin{array}{cc} \frac{Q_{0}}{4\pi D}\left(E_{1}\left(\frac{∥r\;-\;r_{i}{∥}_{2}^{2}}{2D_{s}+4D\left(t-t_{i}\right)}\right)\right. & \\ \left.-E_{1}\left(\frac{∥r\;-\;r_{i}{∥}_{2}^{2}}{2D_{s}+4D\left(t-\left(t_{i}+\tau_{i}\right)\right)}\right)\right), & \mathrm{if}\;r\neq r_{i},\\ \frac{Q_{0}}{4\pi D}\ln\left(\frac{D_{s}\;+\;2D\left(t-t_{i}\right)}{D_{s}\;+\;2D\left(t-t_{i}-\tau_{i}\right)}\right), & \mathrm{if}\;r=r_{i}, \end{array}\right.$$

and when the beam is off, that is, $t>t_{i}+\tau_{i}$. In this paper, for convenience, we also use the following compact form to denote the diffusion distribution:

(15) $$\phi_{i}\left(r,t\right)=\left\{\begin{array}{cc} 0, & \mathrm{for}\;0\leq t<t_{i},\\ \phi_{i}^{\mathrm{on}}\left(r,t\right), & \mathrm{for}\;t_{i}\leq t\leq t_{i}+\tau_{i},\\ \phi_{i}^{\mathrm{off}}\left(r,t\right), & \mathrm{for}\;t_{i}+\tau_{i}<t. \end{array}\right.$$

From Equations (13) and (14) and using L'Hôpital's rule, we have

(16) $$\begin{array}{ccc} \lim_{D\to 0^{+}}\phi_{i}^{\mathrm{on}}\left(r,t\right) & = & \frac{Q_{0}\left(t-t_{i}\right)}{2\pi D_{s}}e^{-\frac{∥r\;-\;r_{i}{∥}^{2}}{2D_{s}}}\;\mathrm{and}\;\lim_{D\to 0^{+}}\phi_{i}^{\mathrm{off}}\left(r,t\right)\\ & = & \frac{Q_{0}\tau_{i}}{2\pi D_{s}}e^{-\frac{∥r\;-\;r_{i}{∥}^{2}}{2D_{s}}} \end{array}$$

and

(17) $$\lim_{D\to \infty }\phi_{i}^{\mathrm{on}}\left(r,t\right)=\lim_{D\to \infty }\phi_{i}^{\mathrm{off}}\left(r,t\right)=0.$$

Equation (16) states that for an asymptotically small diffusion coefficient, the diffusion distribution follows the Gaussian shape of the electron probe with a magnitude that increases proportional to $t-t_{i}$ reaching a maximum at $t=t_{i}+\tau_{i}$. Equation (17) shows that for a large diffusion coefficient, diffusion develops quickly as expected.

In Sections S6 and S7, we highlight important properties of the diffusion distribution in Equation (15). For example, Lemmas S5 and S6 summarise the behaviour of the diffusion distribution as a function of distance to the activation point and predict situations where the diffusion distribution is an increasing, decreasing, convex, or concave function of distance to the activation point. Lemmas S11 and S12 provide similar analyses for diffusion distribution with respect to time. From those findings, we report the following result.

Corollary 1 From Lemmas S11 and S12, for a single electron probe in STEM, at a given time instance, the maximum distribution of diffusing substances happens at the activation point. Moreover, the Maximum Beam Diffusion Distribution (M‐BDD), denoted by $A_{\mathrm{bdd}}^{\max}$, occurs at the activation point at the end of the activation time. Mathematically,

(18) $$\begin{array}{ccc} \phi_{i}\left(r,t\right) & \leq & \phi_{i}\left(r_{i},t\right)\leq \phi_{i}\left(r_{i},t_{i}+\tau_{i}\right)=A_{\mathrm{bdd}}^{\max}\\ & ≔ & \frac{Q_{0}}{4\pi D}\ln\left(1+2\rho^{-1}\tau_{i}\right),\;\mathrm{for}\;r\in R^{2}\;\mathrm{and}\;t\geq t_{i}, \end{array}$$

where $\rho ≔\frac{D_{s}}{D}$ is the ratio between the square width of the Gaussian‐shaped probe and the diffusion coefficient.

### 3.2 Diffusion distribution during STEM acquisition

Having formulated the diffusion distribution activated from an electron probe at a single location, we now focus on the full STEM acquisition.

Our model is based on a STEM acquisition where N spatial locations collected in a set of probe locations $R≔\{r_{1},\text{…},r_{N}\}$ are sequentially scanned with an arbitrary trajectory. Let $T_{s}≔\left\{t_{1},\text{…},t_{N}\right\}$ and $T_{\tau }≔\left\{\tau_{1},\text{…},\tau_{N}\right\}$ be, respectively, the set of activation and dwell times for these positions. Using Equation (15), the *Cumulative Diffusion Distribution (CDD)* from j scanned electron probe positions, for j∈{1,…,N}, is

(19) $$\psi_{j}\left(r,t\right)≔\sum_{i=1}^{j}\phi_{i}\left(r,t\right),$$

or equivalently,

(20) $$\psi_{j}\left(r,t\right)=\left\{\begin{array}{cc} 0, & t<t_{1},\\ \phi_{1}^{\mathrm{on}}\left(r,t\right), & t_{1}\leq t<t_{1}+\tau_{1},\\ \phi_{1}^{\mathrm{off}}\left(r,t\right), & t_{1}+\tau_{1}\leq t<t_{2},\\ \phi_{2}^{\mathrm{on}}\left(r,t\right)+\phi_{1}^{\mathrm{off}}\left(r,t\right), & t_{2}\leq t<t_{2}+\tau_{2},\\ \vdots & \vdots \\ \phi_{j}^{\mathrm{on}}\left(r,t\right)+\sum_{i=1}^{j-1}\phi_{i}^{\mathrm{off}}\left(r,t\right), & t_{j}\leq t<t_{j}+\tau_{j},\\ \sum_{i=1}^{j}\phi_{i}^{\mathrm{off}}\left(r,t\right), & t_{j}+\tau_{j}\leq t\leq t_{j+1}. \end{array}\right.$$

Equation (20) assumes a general STEM configuration with variable dwell and settling times for every electron probe position and an arbitrary scanning probe trajectory. In practice STEM often operates with a constant dwell time and close to zero settling time, that is, for all i∈{1,…,N},

(21) $$\begin{array}{c} \tau_{i}=\tau \;\mathrm{and}\;t_{i+1}=t_{i}+\tau_{i}. \end{array}$$

Equation (20) is applicable to any space filling scanning probe trajectory such as raster, snake, Hilbert,^34^ Z‐order, alternating, spiral^46^, ^47^ and random, which can all be defined by simply considering an appropriate ordering of probe locations within the probe location set R.

In the numerical simulations in Section 6, we show examples of raster, snake, random and alternating scans.

Our analysis of the role of diffusion coefficient on the diffusion distribution caused by a single scanned electron probe position in Equation (16) can be extended to the diffusion distribution in a full STEM acquisition. From Equations (16) and (19), we can compute

(22) $$\begin{array}{ccc} \lim_{D\to 0^{+}}\psi_{j}\left(r,t\right) & = & \lim_{D\to 0^{+}}\phi_{j}\left(r,t\right)+\sum_{i=1}^{j-1}\lim_{D\to 0^{+}}\phi_{i}\left(r,t\right)\\ & = & \frac{Q_{0}\left(t-t_{j}\right)}{2\pi D_{s}}e^{\frac{-∥r-r_{j}{∥}^{2}}{2D_{s}}}+\frac{Q_{0}}{2\pi D_{s}}\sum_{i=1}^{j-1}\tau_{i}e^{\frac{-∥r-r_{i}{∥}^{2}}{2D_{s}}}, \end{array}$$

which shows that for an asymptotically small diffusion coefficient, the CDD will be a sum of Gaussian functions centered at specific probe locations with time‐invariant widths.

## 4 DAMAGE AS A DIFFUSION MECHANISM IN STEM

The CDD in Equation (20) does not model the physical processes underlying the damage induced by electron beam in a sample. We recall from Section 1 that our hypothesis is that sample damage is a function of CDD, without specifying the exact physical mechanisms of damage. We subsequently refer to this generically as *Diffusion Induced Damage (DID)*. In this section we show that the proposed diffusion model can be coupled to any physical damage mechanism that is induced by a diffusion process.

Let λ≥0 be a DID threshold and let $\Lambda_{j}\left(r,t;\lambda \right)$ denote the DID w.r.t to the jth activated probe at a given location r and time t. Therefore, $\Lambda_{j}\left(r,t;\lambda \right)$ can be related to the CDD $\psi_{j}\left(r,t\right)$ in Equation (20) using a (temporal) integration, a nonlinear activation function g, and a pupil function p as

(23) $$\begin{array}{ccc} & & \left(\mathrm{Irreversible}\;\mathrm{DID}\;\mathrm{model}\right)\;\Lambda_{j}\left(r,t;\lambda \right)\\ & & \;≔\int_{t_{1}}^{t}p\left(r,t^{\prime }\right)\cdot g\left(\psi_{j}\left(r,t^{\prime }\right)-\lambda \right)\;dt^{\prime }. \end{array}$$

Equation (23) describes different models for DID. In Equation (23), the non‐linear function g determines how damage is related to the CDD at a given location in space and time and the pupil function p controls how damage at one location impacts damage at other locations. Moreover, integrating the damage quantity over time ensures that the damage is irreversible.

We note that the defined damage threshold controls the amount of acceptable DID, which is related to experimental observations of damage reduction by controlling the temporal distribution of the fluence in STEM.^21^

Depending on the nature of the DID, a suitable activation function g can be defined to represent the important features of the damage. As examples, we provide two illustrations:

- *(DID frequency)* In cases where the frequency of the DID event during STEM acquisition is critical, a sign function can be used:
(24) $$g\left(x\right)=\mathrm{sign}\left(x\right)≔\left\{\begin{array}{cc} 1, & x\geq 0,\\ 0, & x<0. \end{array}\right.$$
- *(DID intensity)* The intensity of the damage can alternatively be defined using a Rectified Linear Unit (ReLU) function:
(25) $$g\left(x\right)=\mathrm{ReLU}\left(x\right)≔\left\{\begin{array}{cc} x, & x\geq 0,\\ 0, & x<0. \end{array}\right.$$

There is also a sensitive feature in the damage model Equation (23) that is accounted for by the use of the pupil function. As an illustration, we consider the following two specific cases for the observation of DID:

- *(Offline DID)* This case accounts for damage that occurs in a location even after that location is scanned. In this case
(26) p(r,t)=1,∀rand∀t.
  Hence, this type of damage can only be observed by re‐scanning the sample provided that re‐scanning does not create further damage. This damage type is referred to as ‘damage after scan’ in Ref. [33].
- *(Online DID)* This case, which is referred to as ‘damage during scan’ in Ref. [33], considers damage that occurs during the irradiation of a given location by the probe. Any damage created in that position after moving probe is not further considered. However, this example requires a careful definition of the pupil function. For example, assuming
(27) $$p\left(r,t\right)=\left\{\begin{array}{cc} 1, & \mathrm{if}\;\exists j\;s.t.\;t_{j}\leq t\leq t_{j+1},\mathrm{and}\;\mathrm{if}\;\exists \\ & \;i>j\;s.t.\;∥r\;-\;r_{i}{∥}_{2}\leq r_{p},\\ 0, & \mathrm{otherwise}. \end{array}\right.$$
  This pupil function states that when the jth position is activated, the value of the damage will increase at a location r only if that location is within a radius $r_{p}$ of at least one of the probes positions that is activated in the remaining scan.

From Equation (23), regardless of the choice of activation and pupil functions, the overall point‐wise DID quantity can be computed at the end of the acquisition as:

(28) $$\Lambda \left(r;\lambda \right)≔\Lambda_{N}\left(r,t_{N}+\tau_{N};\lambda \right),$$

as well as the overall DID as Λ(λ)≔∫Λ(r;λ)dr.

We recall that our goal is to show that the proposed framework of diffusion distribution can be used in conjunction with a damage mechanism. However, the feasibility of Equations (23) and (28) depends on the accuracy of the damage activation and pupil functions, as well as on the damage threshold. Hence, we require a quantity that is agnostic to any damage related assumptions so that different systems can be compared only based on variations on models of diffusion distribution.

We note that the CDD $\psi_{j}\left(r,t\right)$ does not incorporate sufficient information about DID, since the DID may happen at any time prior to the time the CDD is computed. Hence, we define a quantity, which can be used as an indicator of a DID event, the maximum value of the CDD at every spatial point taken over all previous time instances. More formally, we introduce the Point‐wise Maximum Cumulative Diffusion Distribution (PM‐CDD) w.r.t. to the jth electron probe position, as $\chi_{j}\left(r,t\right)$ and defined as

(29) $$\chi_{j}\left(r,t\right)≔\max_{\begin{array}{c} 1\leq j^{\prime }\leq j\\ t_{j^{\prime }}\leq t^{\prime }\leq t \end{array}}\;\psi_{j^{\prime }}\left(r,t^{\prime }\right),\;\mathrm{for}\;t\geq t_{j}.$$

The benefit of introducing PM‐CDD is twofold. First, in the case of unknown damage threshold and activation and pupil functions, PM‐CDD can be used to compare different STEM scans and provide information about how likely the DID can occur. Second, the design of DID‐free STEM scans, discussed in Section 5.1, will involve a direct comparison of the PM‐CDD with a damage threshold, which is computationally less demanding compared to the use of CDD.

We introduce a reduced and simplified notation, where if the index of the electron probe position is not specified, the term PM‐CDD refers to the PM‐CDD at the end of the scan as:

(30) $$\chi \left(r\right)≔\chi_{N}\left(r,t_{N}+\tau_{N}\right).$$

Therefore, as mentioned above, the PM‐CDD provides information about the likelihood of DID occurring during the full scan regardless of the damage mechanism. Subsequently in Section 5.1, we will use the PM‐CDD in a comparison of different STEM scans.

Furthermore, from the PM‐CDD, we can define a Global Maximum of Cumulative Diffusion Distribution (GM‐CDD) $\chi^{\max}$, as

(31) $$\chi^{\max}≔\max_{r}\chi \left(r\right),$$

which gives the maximum value of the CDD over all spatial and temporal points during an acquisition. This value can be used to identify a DID‐free STEM scan that is agnostic to the DID model. We define a STEM scan to be DID‐free, if the overall DID quantity is zero, that is, Λ(λ)=0. The following theorem, which is proved in Section S8, relates the DID profile to the GM‐CDD.

Theorem 1 Consider a DID model characterised by a non‐negative activation function g, that is $g:R\mapsto R_{\geq 0}$, in either Equation (24) or Equation (25), and a pupil function p, that is, p:R↦R, such that $\int_{t_{1}}^{t_{N}}p\left(r,t^{\prime }\right)\;dt^{\prime }>0,\forall r$. A STEM scan is DID‐free, iff the GM‐CDD is less than or equal to the DID threshold. Formally,

(32) $$\left(\mathrm{DID}-\mathrm{free}\;\mathrm{STEM}\right)\;\Lambda \left(\lambda \right)=0,\;⟺\;\chi^{\max}\leq \lambda .$$

Theorem 1 shows that for a DID‐free STEM scan, it is only necessary to examine the GM‐CDD value. Hence, that quantity, or the PM‐CDD, will be used as a measure in a comparison of different STEM scans in Section 6.4.

## 5 DIFFUSION DISTRIBUTION IN COMPRESSIVE STEM

To reduce sample damage in STEM, several alternative (nonraster) scans^25^, ^48^, ^49^ have been proposed based on CS^23^, ^24^ which have demonstrated that sample damage can be reduced or mitigated. A common theme in these studies is to activate the electron probe at a small subset of possible locations, that is, to subsample probe locations, and to recover a STEM image from those incomplete measurements by solving an inverse problem, known as inpainting.

The CDD and DID formulated in Equations (20) and (23), respectively, can be simply adapted for compressive STEM scans. Let $s≔{\left[s_{1},\text{…},s_{N}\right]}^{⊤}\in {\left\{0,1\right\}}^{N}$, with $\sum_{j}s_{j}=M\ll N$, be a binary vector associated with a subsampling of M probe locations; $s_{j}=1$ if the electron probe is activated at the jth location $r_{j}$ and $s_{j}=0$, if it is not activated. From Equation (19), the CDD in compressive STEM w.r.t. to the subsampling strategy s is given as

(33) $$\psi_{j}^{\mathrm{cs}}\left(r,t\right)≔\sum_{i=1}^{j}s_{i}\phi_{i}\left(r,t\right).$$

We note that Equation (33) supports any arbitrary subsampling strategy of probe positions. By comparing the DID profile for full STEM, that is, Λ(r;λ), and compressive STEM, that is, $\Lambda^{\mathrm{cs}}\left(r;\lambda \right)$, it is clear that compressive STEM has the advantage of a lower DID. Alternatively, since the PM‐CDD in compressive STEM is less than the PM‐CDD in full STEM,

$$\Lambda^{\mathrm{cs}}\left(r;\lambda \right)\leq \Lambda \left(r;\lambda \right).$$

Direct application of Equation (33) allows the modelling of a compressive STEM scan using a beam blanker to perform probe subsampling.^50^ Alternatively for the use of a programmable scan generator, Equation (19) can be used to redefine the set of scanned probe locations R and associated activation times $T_{s}$. We also recall that the acquisition time in compressive STEM with a beam blanker and a scan generator is proportional to, respectively, the number of total probe positions and subsampled probe positions.

From the findings in Corollary1, we derive the following theorem, which states the sufficient condition in compressive STEM for reducing DID.

Theorem 2 Let λ>0 be the DID threshold of a sample and $\tau^{\max}≔\max_{i}\tau_{i}$ the longest dwell time of the electron probe. Therefore, if

(34) $$A_{\mathrm{bdd}}^{\max}=\frac{Q_{0}\rho }{2}\ln\left(1+2\rho^{-1}\tau^{\max}\right)\geq \lambda ⟹\Lambda \left(\lambda \right)>0.$$

Hence, if DID is due to an individual electron probe, rather than the CDD, subsampling probe positions using any strategy will not result in a DID‐free STEM.

The total number of diffusing species during the acquisition also needs to be considered when comparing conventional and compressive STEM. The application of Remark 2 to the CDD in Equations (20) and (33) gives the total number of deposited electrons for conventional and compressive STEM, $Q_{\mathrm{stem}}$ and $Q_{\mathrm{cstem}}$, respectively, as

(35) $$Q_{\mathrm{stem}}=Q_{0}\sum_{j=1}^{N}\tau_{j},\;\mathrm{and}\;Q_{\mathrm{cstem}}=Q_{0}\sum_{j=1}^{N}s_{j}\tau_{j}.$$

From the above it is evident that $Q_{\mathrm{cstem}}\leq Q_{\mathrm{stem}}$; fewer units are diffused in compressive STEM compared to conventional STEM as intuitively expected. In a simplified model with a constant dwell time for all electron probe positions, that is, $\tau =\tau_{j}$ for all j∈1,…,N, these quantities become

(36) $$Q_{\mathrm{stem}}=NQ_{0}\tau ,\;\mathrm{and}\;Q_{\mathrm{cstem}}=MQ_{0}\tau .$$

### 5.1 Diffusion controlled sampling strategy for DID‐free compressive STEM

As described above, compressive STEM results in a lower DID compared to conventional STEM. However, this raises the following questions:

(i) Given a DID threshold for constraining the PM‐CDD, what is the optimal design for subsampling mask containing the maximum number of probe locations?
(ii) Given a fixed number of scanned probe locations, what is the optimal design of a mask minimising DID or PM‐CDD?

Finding a general subsampling strategy for both cases will require advanced tools from optimisation theory and is deferred to a subsequent publication. Hence, in this paper we confine ourselves to providing a solution to the first question only.

Our approach for designing a Diffusion Controlled Sampling (DCS) strategy of the probe positions that results in DID‐free STEM is as follows; given a DID threshold λ as in Equation (28), a probe location in the subsampling mask R is selected, only if the PM‐CDD w.r.t. to that electron probe over all spatial locations is lower than that DID threshold as

(37) $$j\in R,\;\mathrm{if}\;\chi_{j}\left(r,t_{j}+\tau_{j}\right)<\lambda ,\;\mathrm{for}\;\mathrm{all}\;r.$$

Using Theorem 1, this approach guarantees that the GM‐CDD $\chi^{\max}<\lambda$ and hence, $\Lambda^{\mathrm{cs}}\left(r;\lambda \right)=0$ for all locations r and DID does not occur. We provide numerical examples of this approach in Section 6.5. We note here that this problem does not necessarily have a unique solution and the approach described provides only one example of such a DCS strategy. However, other approaches, for example, based on constraining the minimum distance between two subsampled electron probes, can be simply incorporated within our approach.

## 6 NUMERICAL RESULTS

In this section, we describe numerical simulations^2^ in support of the analyses above, which highlight important properties of DID in STEM. In the simulations reported, we ignore the effects of sample drift and scan coil dynamics.

At the start of this section we note that despite the availability of a closed‐form solution for CDD in STEM, the associated numerical simulations are still computationally demanding. Therefore, in Section S9, we analyse the time complexity of computing the CDD in Equations (20) and (33) for full STEM and compressive STEM, respectively. We also report approximation alternatives for accelerated simulation followed by numerical confirmations.

**Baseline STEM**. The parameters for the baseline simulation are given in Table 1. Subsequently, for other simulations, we report only those parameters that differ from this baseline setup. We consider a square array of probe positions giving $N=20^{2}$ total probes. We further assume a conventional STEM scan described by Equation (21) with a constant dwell time and zero settling time. This equates to a 4ms acquisition time and 0.1nm probe radius. The diffusion coefficient in Table 1 is in the range of the values that was studied in Refs. 33 and 21 for a specific zeolite sample. This setting is equivalent to M‐BDD $A_{\mathrm{bdd}}^{\max}=10^{4}\;u\cdot {\mathrm{nm}}^{-2}$ and a total number of diffusing species $Q_{\mathrm{stem}}=253.83$ Ku. We now let $R_{\mathrm{sim}}$ be a set of spatial points defined as the simulation grid. We assume that $N_{T}$ temporal grids are defined during the scan of every two consecutive electron probe positions, that is, $t_{i}\leq t\leq t_{i+1}$. Each scan step length is simulated using 10 pixels, that is, $|R_{\mathrm{sim}}|=100N$. Furthermore, only time instances w.r.t to the probe dwell times are simulated, that is, $N_{T}=1$. Simulations of the diffusion distribution for this baseline STEM scan on a personal computer equipped with an Intel(R) Core(TM) i7 CPU took approximately 50 min.

**TABLE 1:** Parameter setting for a baseline STEM scan. A 20×20 square grid is considered for a raster scan of probe positions. These equate to a 4ms acquisition time and 0.1nm probe radius.

Parameter	Value	Parameter	Value
Dwell time	τ=10μs	Scan step size	$\Delta_{p}=0.05\;\mathrm{nm}$
Settling time	$\bar{\tau }=0\;s$	diffusion coefficient	$D=10{\;\mathrm{nm}}^{2}\cdot s^{-1}$
Probe width parameter	$D_{s}=0.01{\;\mathrm{nm}}^{2}$	Initial deposited units	$Q_{0}=63.45\;Mu\cdot s^{-1}$

### 6.1 Diffusion distribution in a full STEM scan

**FIGURE 3:**
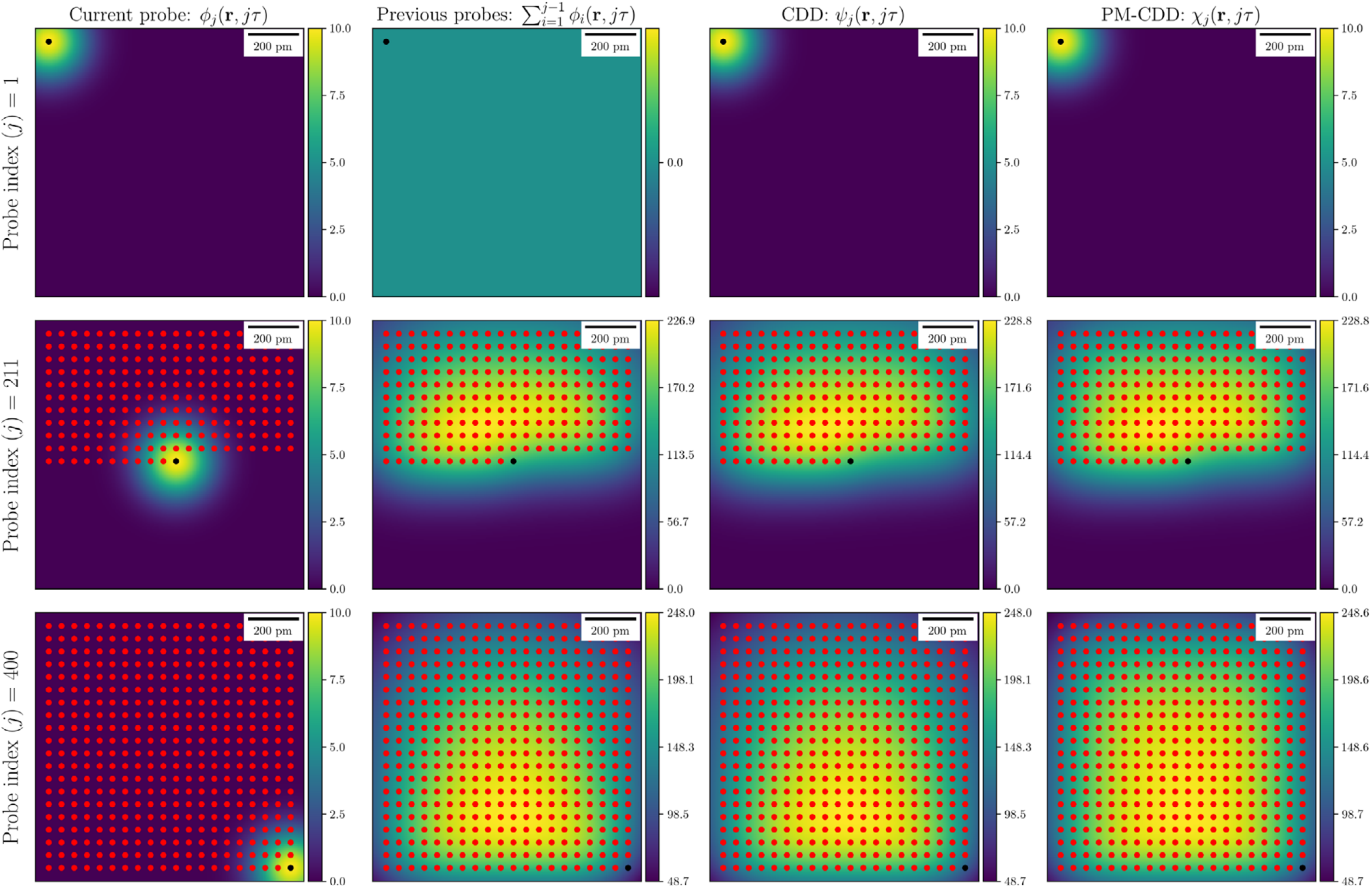
Snapshots of diffusion distribution in a baseline STEM scan for a probe activated at three selected positions. Top, middle and bottom rows correspond to the first, middle and last activated probe, respectively. The first column is the diffusion distribution caused by the electron probe activated at the current position. The second column shows the cumulative diffusion distribution caused by the electron probe activated at the previous location. The third column is the sum of the first two columns. The last column is the point‐wise maximum of the cumulative diffusion distribution. Red and black points denote, respectively, the previously and currently activated electron probe positions. Every probe has an M‐BDD $A_{\mathrm{bdd}}^{\max}=10^{4}\;u\cdot {\mathrm{nm}}^{-2}$, although the PM‐CDD, which controls the DID, is 24.8 times greater. The units of colour bars are $10^{3}\;u\cdot {\mathrm{nm}}^{-2}$.

We first simulate the CDD for a full STEM scan using the parameters in Table 1. Figure 3 illustrates (i) the diffusion distribution $\phi_{j}$ of the activated electron probe at the first, middle and last locations (first column), (ii) the CDD of previously scanned electron probe positions $\sum_{i=1}^{j-1}\phi_{i}$ (second column), (iii) CDD $\psi_{j}$ w.r.t to the jth electron probe position (third column), and (iv) the corresponding PM‐CDDs $\chi_{j}$, during a full STEM scan, for three representative activated electron probe positions (last column). We use Equation (20) for (i–iii) and Equation (29) for (iv). Figure 3 shows that although the M‐BDD $A_{\mathrm{bdd}}^{\max}=10^{4}\;u\cdot {\mathrm{nm}}^{-2}$, the PM‐CDD χ(r) reaches a maximum value of $248.6\cdot 10^{3}\;u\cdot {\mathrm{nm}}^{-2}$, which determines the DID.

**FIGURE 4:**
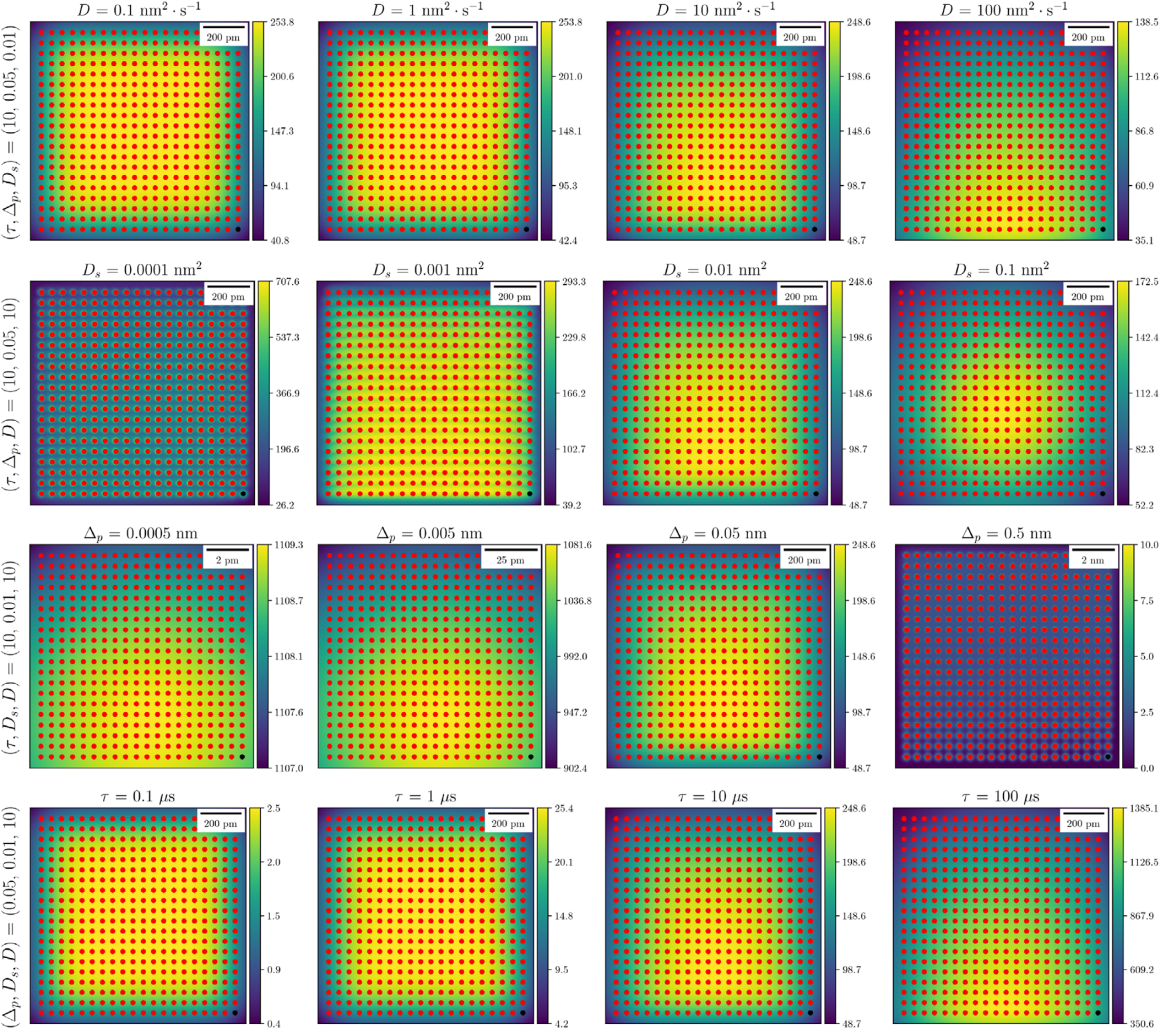
Comparison of PM‐CDD χ(r) for different STEM scans. Diffusion coefficient (first row), probe width parameter (second row), scan step (third row), and dwell time (fourth row) are varied. The PM‐CDD of a STEM scan increases by either decreasing the diffusion coefficient, increasing the probe radius, decreasing the scan step size, or increasing the dwell time. Other parameters are the same as in Table 1. The total number of deposited diffusing units is constant in the systems shown in the first three rows, that is, $Q_{\mathrm{stem}}=253.8$ Ku, whereas in the fourth row, it is proportional to the dwell time. The units of colour bars are $10^{3}\;u\cdot {\mathrm{nm}}^{-2}$.

In Figure 4, we show an extended analysis with additional simulations for multiple values of the diffusion coefficient $D\in \left\{0.1,1,10,100\right\}\;{\mathrm{nm}}^{2}\cdot s^{-1}$, probe width parameter $D_{s}\in \left\{10^{-4},10^{-3},10^{-2},10^{-1}\right\}\;{\mathrm{nm}}^{2}$, scan step size $\Delta_{p}\in \left\{0.0005,0.005,0.05,0.5\right\}\;\mathrm{nm}$, dwell time τ∈{0.1,1,10,100}μs. Figure 4 compares the PM‐CDD of these systems.

From Figure 4 we observe that the PM‐CDD increases if (i) the diffusion coefficient decreases, (ii) the probe radius increases, (iii) the scan step size decreases, or (iv) the dwell time increases. Moreover as we showed in Equation (22) for a very small diffusion coefficient, the PM‐CDD reduces to a sum of Gaussian functions with constant width parameter $D_{s}$ consistent with this behaviour. In the first row of Figure 4, it is clear that for a diffusion coefficient less than $D=10{\;\mathrm{nm}}^{2}\cdot s^{-1}$, the PM‐CDD stays almost constant and the resulting heatmap is a sum of Gaussian functions. For a very small probe radius, the Gaussian‐shaped electron probe approximates to a point like source, which is evident in the second row of Figure 4. However, a very small probe radius is functionally equivalent to very distant probe locations. Hence, the two heatmaps in Figure 4 corresponding to $D_{s}=0.0001{\;\mathrm{nm}}^{2}$ and $\Delta_{p}=0.5\;\mathrm{nm}$ are similar in shape but are significantly different in their range of the values. We recall that in the work described here the total number of diffusing units in the system does not depend on the probe radius; hence, reducing the probe to a point source simply amounts to a very high value of the diffusion distribution at the scanned probe positions.

We emphasise that our aim in simulations is to illustrate the variability of the distribution of cumulative diffusion rather than to propose a specific experimental damage reduction strategy. In this regard we further note that (i) the diffusion coefficient is sample‐dependant; (ii) increasing the probe radius reduces the image resolution in STEM; (iii) increasing the electron probe step size also reduces the image resolution in STEM; and (iv) reducing the dwell time can result in scan distortions and reduced signal‐to‐noise ratio.^51^

**FIGURE 5:**
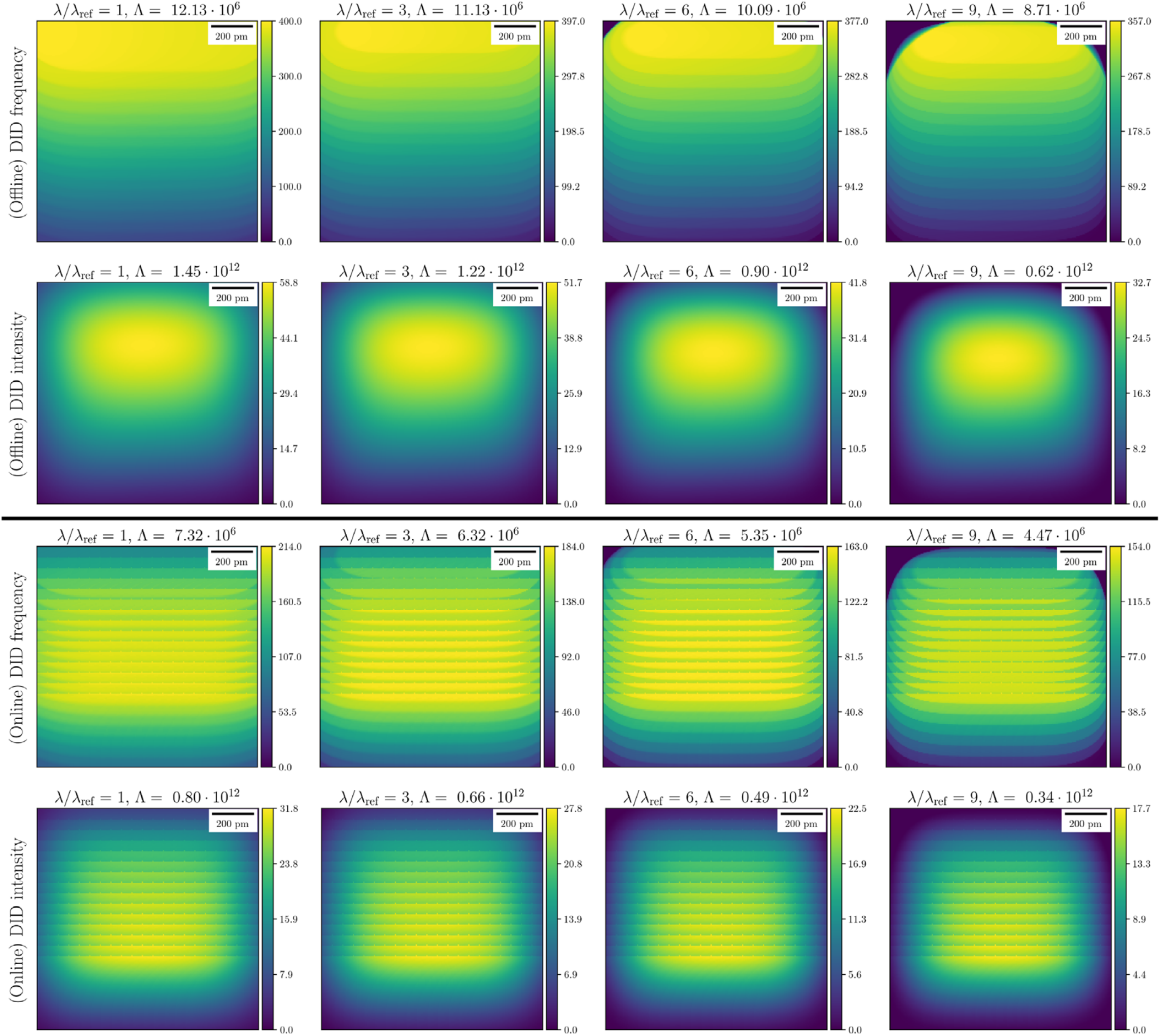
DID profiles Λ(r;λ) with offline (top) and online (bottom) observations for different damage thresholds. The units of colour bars and Λ for DID are $u\cdot {\mathrm{nm}}^{-2}$, with colour bars scaled by a factor $10^{6}$.

Different DID models are illustrated in Figure 5 for the baseline STEM scan. We have chosen the models characterised by the sign and reLU activation functions in Equations (24) and (25), respectively. Both offline and online observations of DID with a pupil function given by Equations (26) and (27), respectively, are considered. For the former we set $r_{p}=3\cdot \Delta_{p}$. We define $\lambda_{\mathrm{ref}}≔A_{\mathrm{bdd}}^{\max}=\frac{Q_{0}\rho }{2}\ln\left(1+2\rho^{-1}\tau \right)$, that is, $\lambda_{\mathrm{ref}}=10^{4}\;u\cdot {\mathrm{nm}}^{-2}$ in this example to be the reference quantity for the DID threshold. We subsequently simulated the DID profiles for different DID thresholds λ, such that $\lambda /\lambda_{\mathrm{ref}}\in \left\{1,3,6,9\right\}$.

Figure 5 shows that the choice of both activation and pupil functions significantly changes the shape of the DID profile, which emphasises the importance of accurate modelling of damage mechanisms. Offline DID would be observed if the sample was re‐scanned and that damage is exacerbated in the upper part of the FoV, since this region has experienced more DID during the full acquisition. In contrast, online DID is observed during the scan and since damage will be recorded only at locations that will be scanned later on, the lower part of the FoV shows higher DID quantities. Together, these results imply that even if DID is not observed during a scan, it may have occurred at previously irradiated locations in the sample. The discontinuity observed in the online DID maps is the effect of the pupil function.

### 6.2 Impact of a blanking time on diffusion distribution

As discussed in Section 6.1, decreasing the dwell time has a substantial impact in reducing the PM‐CDD and DID, but at a potential cost of increased scan distortions. One way to mitigate DID is by allowing the diffusion process to dissipate (or equivalently, allowing the sample to relax) between scan positions. This can be achieved by blanking and unblanking the electron beam^26^, ^52^ at every probe positions with a specific duty cycle, which can also reduce the scan distortions. Importantly, using this approach the number of deposited electrons in the system and hence, the signal‐to‐noise‐ratio, remains unchanged.

**FIGURE 6:**
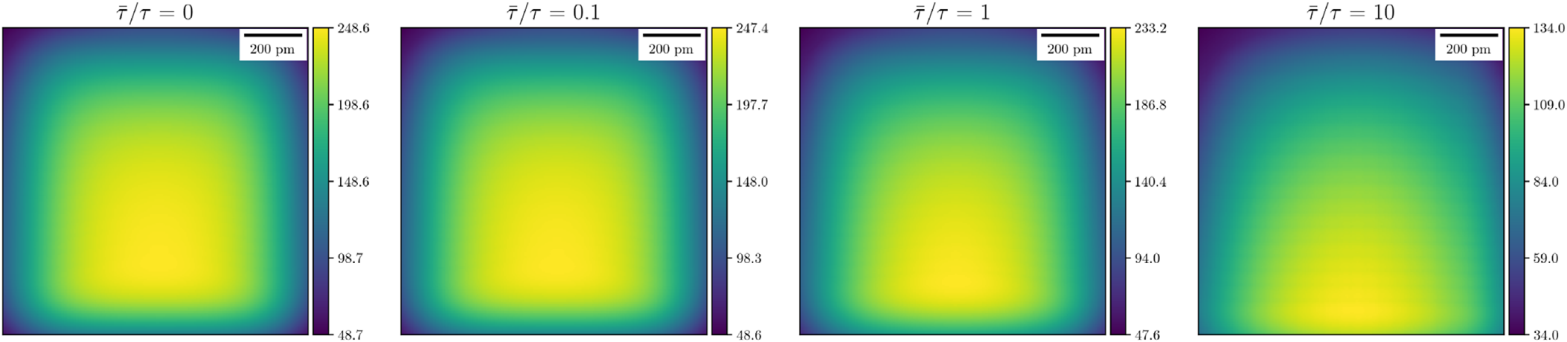
STEM scans with identical total number of diffusing species but different blanking times. PM‐CDDs χ(r) given by Equation (29) are shown. Increasing the blanking time reduces the PM‐CDD. The total number of diffusing units in all four systems is $Q_{\mathrm{stem}}=253.83\;\mathrm{Ku}$. The units of colour bars are $10^{3}\;u\cdot {\mathrm{nm}}^{-2}$.

Our framework can accommodate beam blanking through consideration of the settling time in Equation (20). To further explore this, we let $\bar{\tau }$ be a constant blanking time for every probe position, that is, in Equation (20) we set $\bar{\tau }=t_{j}-\left(t_{j-1}+\tau_{j-1}\right)$ for all j∈{2,…,N}. Figure 6 shows the PM‐CDD maps for STEM scans with different ratios of blanking to dwell time $\bar{\tau }/\tau \in \left\{0,0.1,1,10\right\}$. We note that $\bar{\tau }/\tau =0$ is baseline STEM. As expected, increasing the blanking time reduces the PM‐CDD, for a constant total number of diffusing units.

### 6.3 Impact of the scan trajectory on the diffusion distribution

The discussion in Section 1 showed that changing the sequence of scanned probe positions ^21^ is a useful approach for damage reduction.

To support this hypothesis we have simulated the PM‐CDD for different scanning probe trajectories The values of $Q_{0},D$, $D_{s}$, and $\Delta_{p}$ are identical to those in Table 1.

**FIGURE 7:**
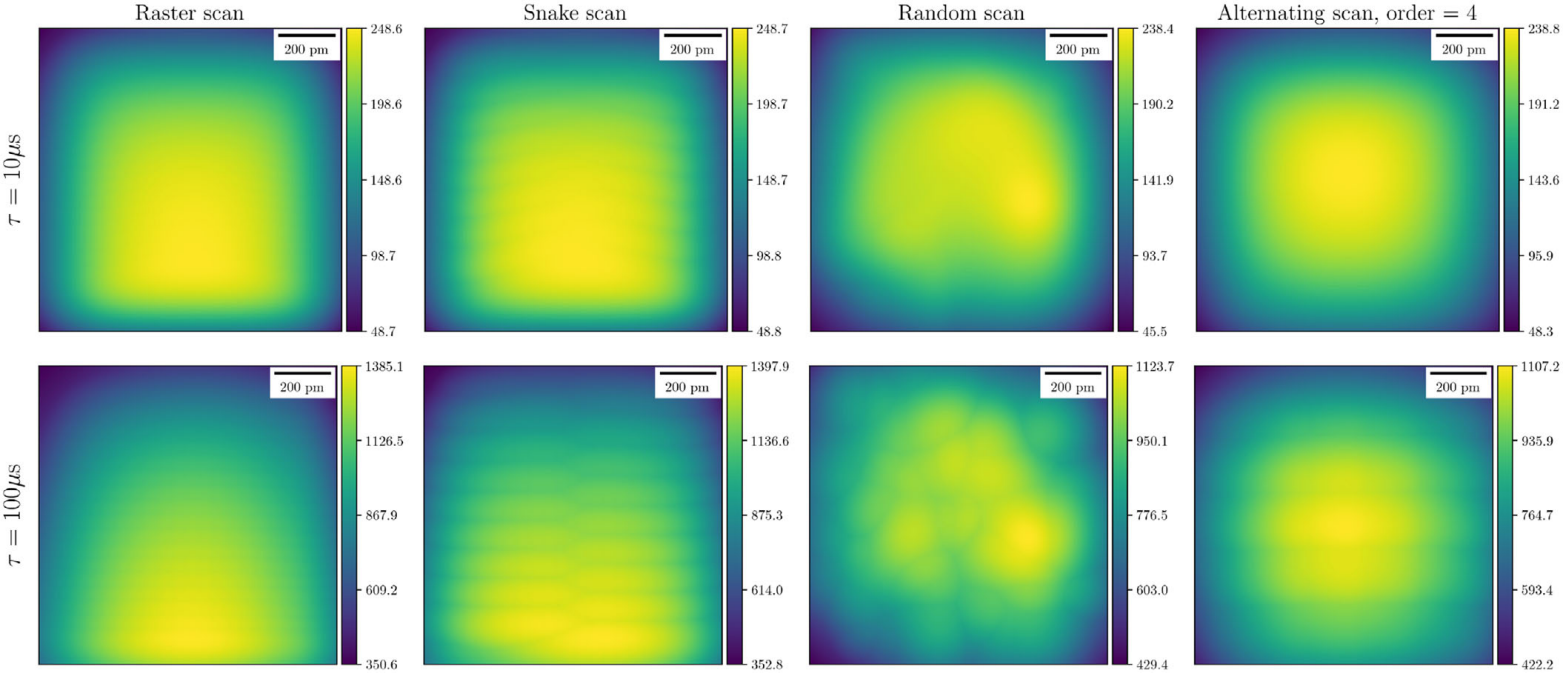
PM‐CDD χ(r) for different scan trajectories. Longer dwell times result in higher PM‐CDD for all four scan trajectories. Random and alternating scans show a reduced PM‐CDD compared to a raster scan, while a snake scan increases the PM‐CDD. The impact of scan trajectory is more evident for longer dwell times. The units of colour bars are $10^{3}\;u\cdot {\mathrm{nm}}^{-2}$.

Figure 7 compares the PM‐CDD for raster, snake, random and alternating scans as defined in Section 3.2 for two values of dwell time τ∈{10,100}μs. As expected, the scan trajectory has a significant impact on the spatial distribution of the PM‐CDD; random and alternating scans also show an overall reduction in the PM‐CDD, that is, less DID, whereas in contrast the snake scan increases the DID.

**FIGURE 8:**
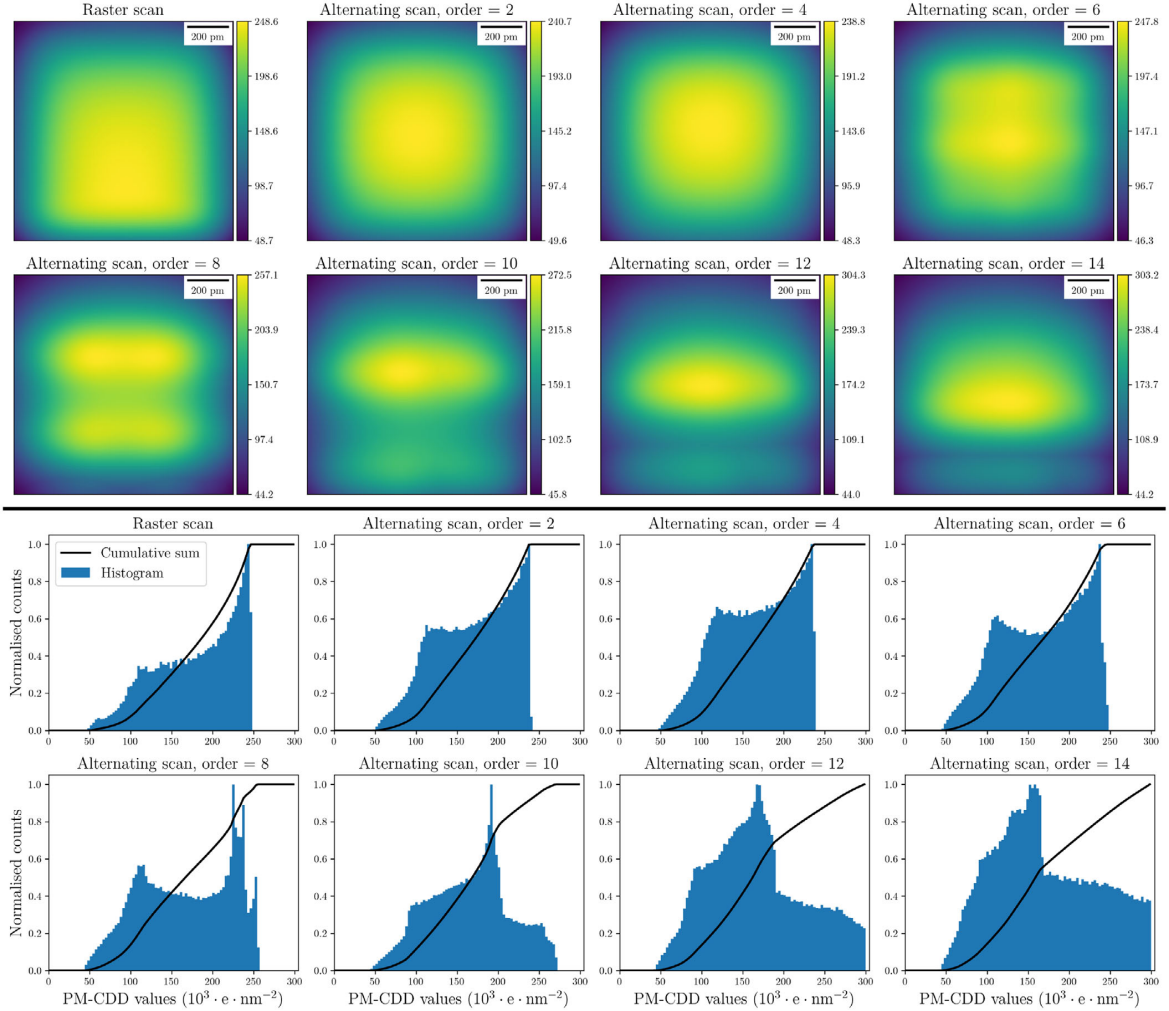
PM‐CDD χ(r) for different orders of alternating scans. Spatial distributions (top) and corresponding histograms (bottom) are plotted. The units of colour bars are $10^{3}\;u\cdot {\mathrm{nm}}^{-2}$.

In Figure 8, alternating scans with different orders κ∈{1,2,4,6,8,10,12,14} and with a dwell time τ=10μ s are shown. An alternating scan of order one is equivalent to a conventional raster scan. However, the PM‐CDD χ(r) is a nonmonotonic function of alternating scan order κ and hence, changing the order of an alternating scan does not necessarily reduce the DID. Figure 8 shows the corresponding normalised histogram of the PM‐CDD, where the shape of the histogram and the cumulative sum of the histogram is affected by the order of the alternating scan. Lower‐order alternating scans, for example, when κ∈{1,2,4}, have a sharp peak in their histograms, which indicates that a substantial portion of sample will be damaged as the PM‐CDD crosses the DID threshold. However, for higher‐order alternating scans the histograms are more uniform. In these cases if a DID occurs it will affect a smaller portion of the sample. Accordingly the slope of the cumulative sum curve can be considered as an indicator of the risk of DID. For example, the slope of the cumulative sum, around $248.62\;Ku\cdot {\mathrm{nm}}^{-2}$, is sharp for a raster scan but is reduced by increasing the order of an alternating scan. Overall, in comparison to a raster scan, an alternating scan with an arbitrary order does not necessarily reduce the maximum value of PM‐CDD but can result in more uniform values of this quantity.

### 6.4 Diffusion distribution in compressive STEM

Compressive STEM is a method for reducing the total fluence deposited over a region of interest without reducing the signal‐to‐noise ratio per measurement. In this section, we simulate the PM‐CDD for compressive STEM following Equation (33), with two subsampling strategies: linehop ^38^ and Uniform Density Sampling (UDS) (subsampling probe positions uniformly at random). For the UDS strategy we consider subsampling of the electron probe positions with either a beam blanker or a scan generator, as described in Section 1. However, we simulate the linehop strategy using only a scan generator, since, for zero settling time, performing a linehop scan with a beam blanker and with a scan generator results in an identical CDD. Other parameters used are the same as those in Table 1.

Our choice to study both a scan generator and beam blanker is motivated as these are the experimental, practical options for subsampling in STEM. However, these two regimes exhibit two distinct timings. Using a beam blanker, time passes while the blanked beam is not scanning. In contrast using a scan generator, the beam is moved immediately and each exposure is followed by another exposure. In both cases considered here, the sample is exposed to the same number of total electrons and the acquisition is considered to be perfect in that there is no delay between the end of a sampling period and the activation of the beam blanker and that there is no hysteresis moving the probe from one location to another.

**FIGURE 9:**
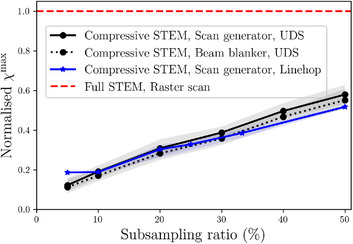
Normalised GM‐CDDs $\chi^{\max}$ in compressive scans. Ten Monte‐Carlo trials over random generation of subsampling masks were performed. Shaded areas represent the variance of normalised GM‐CDDs. Subsampling probe positions using either a linehop or a UDS strategy reduces the GM‐CDD. This reduction is not proportional to the subsampling ratio. The use of a beam blanker can further reduce the GM‐CDD. A Linehop strategy shows a reduction of the GM‐CDD for sampling ratios greater than 10%. Example maps taken from this figure are shown in Figure 10.

**FIGURE 10:**
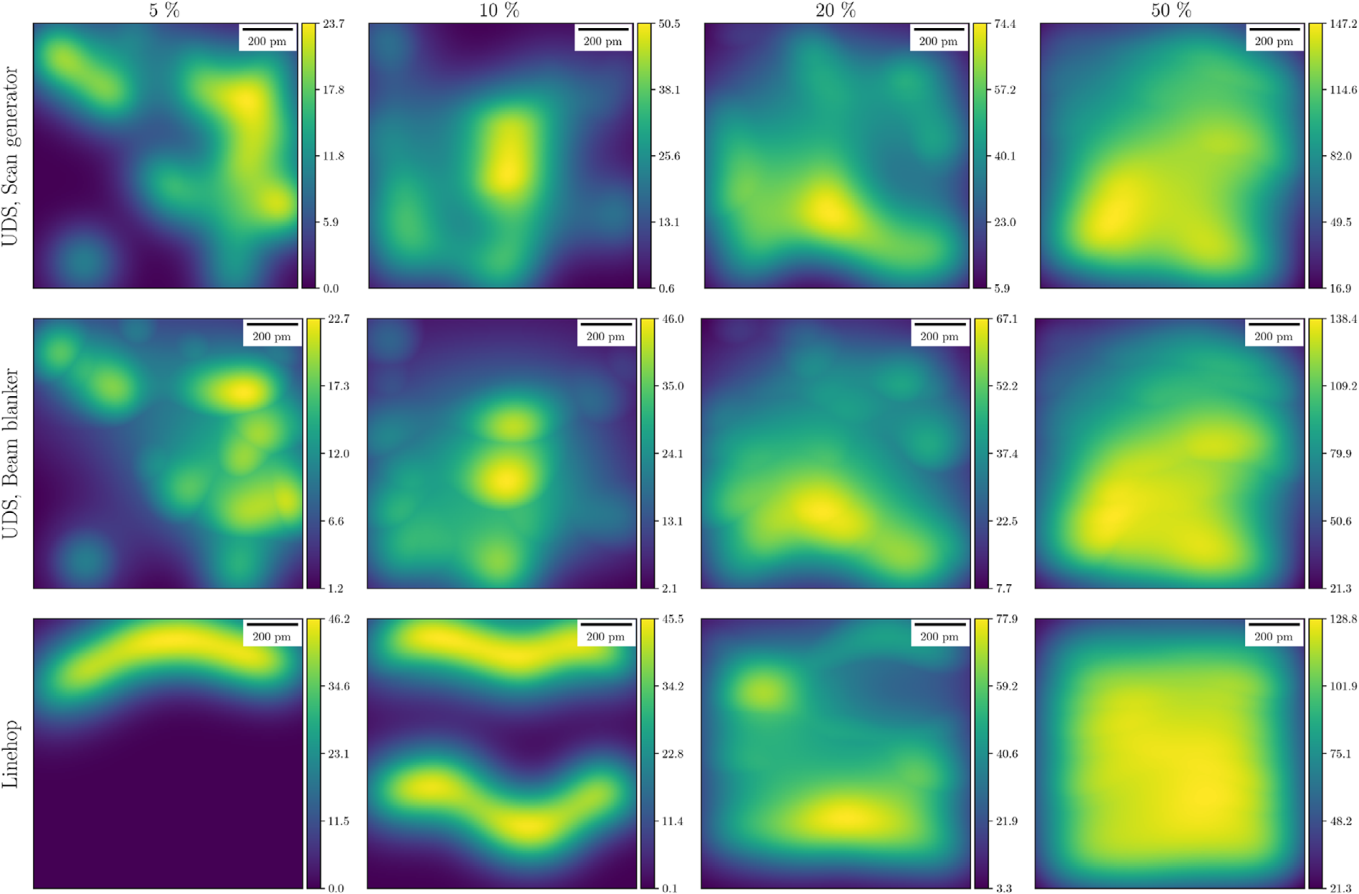
PM‐CDDs χ(r) in compressive STEM scans for different sampling ratios. These are example maps taken from the simulations in Figure 9. The same subsampling mask of electron probe positions is used for both UDS strategies, whereas the use of a beam blanker yields lower GM‐CDD value. The units of colour bars are $10^{3}\;u\cdot {\mathrm{nm}}^{-2}$.

Figure 9 compares the GM‐CDD defined in Equation (31) for a raster scan with two compressive STEM scans, for multiple subsampling ratios. We consider M/N={5,10,20,30,40,50}% for the UDS strategy and M/N={5,10,20,25,33.3,50}% for the linehop strategy. To account for variability in the random generation of subsampling strategies, 10 Monte‐Carlo trials were run for each data point in Figure 9, using different randomly generated subsampling masks. The empirical averages of GM‐CDDs in those trials are shown by lines in Figure 9, while the shaded areas correspond to the variance of GM‐CDDs. All compressive scans yield lower GM‐CDD value compared to those of the raster scan. The GM‐CDD of the UDS strategy is further reduced by the use of a beam blanker. An important observation in Figure 9 is that the ratio between the GM‐CDD for full STEM and compressive STEM is not equal to the subsampling ratio. This indicates that compressive STEM with M/N(%) probes does not reduce the GM‐CDD of the full STEM by the same M/N factor. As an example, a 10% subsampling of the probes reduces the GM‐CDD by only a factor of ≈5. Example CDDs of the experiments in Figure 9 are shown in Figure 10.

### 6.5 Designing DCS masks for DID‐free STEM

In this section, we use the previous results to design DCS subsampling strategies for compressive STEM applications. The STEM parameters in Table 1 are used and we consider two compressive STEM scans using either a scan generator or a beam blanker.

As described in Section 5.1, the compressive framework allows the design of a subsampling mask of probe positions, ensuring that DID is eliminated. To design such a subsampling mask we follow the rule defined by Equation (37); a probe location is selected, only if the corresponding PM‐CDD is less than the DID threshold at every spatial location. Taking $\lambda_{\mathrm{ref}}=10^{4}\;u\cdot {\mathrm{nm}}^{-2}$ from Section 6.1 as the reference quantity for DID threshold, we have designed DCS masks for different DID thresholds λ, such that $\lambda /\lambda_{\mathrm{ref}}\in \left\{1,2,\text{…},25\right\}$. The resultant sampling ratio M/N has been measured after each trial.

**FIGURE 11:**
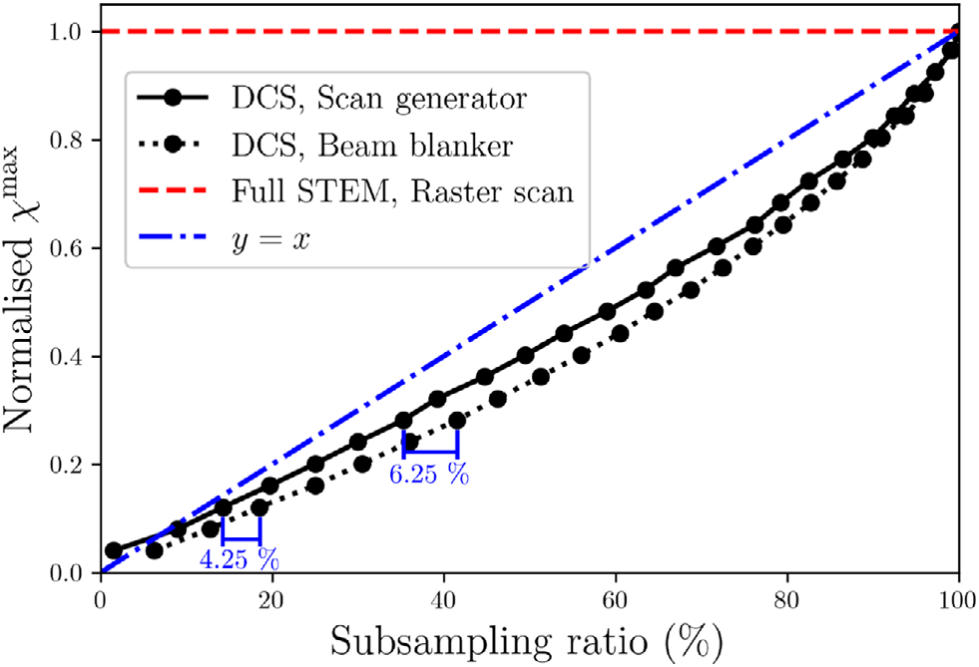
Normalised GM‐CDD values for DCS masks with respect to the damage threshold. The use of a beam blanker improves subsampling efficiency in this experiment by 4–7% for $\lambda /\lambda_{\mathrm{ref}}<10$, or equivalently, for subsampling ratios less than 50%. Example maps taken from this figure are shown in Figure 12.

Figure 11 shows the GM‐CDD, which is constrained by the DID threshold ratio $\lambda /\lambda_{\mathrm{ref}}$, as a function of the subsampling ratio in the designed masks. Comparison of the curves in Figure 11 with the y=x line highlights the efficiency of the DCS strategy and shows that the reduction in relative GM‐CDD is greater than the subsampling ratio. Moreover, we observe that for a DID threshold λ equivalent to a horizontal line in Figure 11, the use of a beam blanker improves the efficiency of the sampling strategy by 4–7% for $\lambda /\lambda_{\mathrm{ref}}<10$. This arises because, in the simplified models used here, a beam blanker allows the system to ‘cool’ or relax between successive subsampled probe positions, while a scan generator instantaneously moves the electron probe to the next sampled location. Furthermore, when comparing the two masks, we note that the acquisition time for compressive STEM with a beam blanker and scan generator is, respectively, Nτ and Mτ s.

**FIGURE 12:**
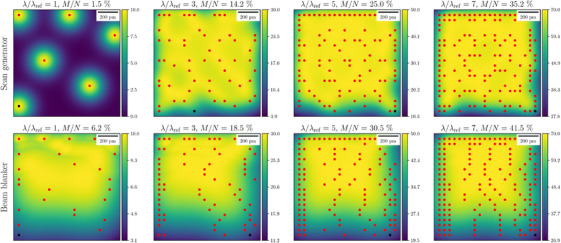
PM‐CDD χ(r) from two DCS masks with different sampling ratios. These are example maps taken from the simulations in Figure 11. The units of colour bars are $10^{3}\;u\cdot {\mathrm{nm}}^{-2}$.

## 7 CONCLUSIONS

We have described a mathematical framework for understanding diffusion distribution in STEM scans. We have derived the first closed‐form formulation of the diffusion distribution for a Gaussian‐shaped electron source, used as a proxy to the airy disc function. Our analyses can be directly applied to 4‐D STEM scans and can be used in various STEM acquisition modalities.

Despite significantly improving both the simulation speed and accuracy of the diffusion process in STEM compared to previous work,^32^, ^33^ numerical simulation within the framework described is limited by the size of the simulation space in turn determined by the size of the electron probe positions grid, and the dimensions of the temporal and spatial grids. Parallel programming or GPU implementation of the simulations can significantly boost the time performance given suitable storage requirements.

In Section 3, we limited our analysis to the role of the diffusion coefficient in the asymptotic regime. A more in‐depth study, for example based on the first and second derivatives of both the diffusion distribution and the CDD, will the subject of future research. We have also limited our discussion in Section 3.1 to studies of the first and second derivatives of a single electron probe diffusion distribution without considering the dynamics of the CDD and PM‐CDD.

Our proposed damage models have yet to be validated using experimental data for which one of the required modifications to our model would be to support a secondary damage mechanism and possibly a healing effect. Moreover, we postulate that the units of the proposed damage model, that is, $u\cdot {\mathrm{nm}}^{-2}$, need to be converted to give a physically tractable unit of damage.

An important future extension of this work would be to develop a framework for diffusion in three dimensions. This will require a derivation of the counterpart of Equation (15) from Equation (3) in 3‐D, together with experimental measurements of damage in the axial direction and the effect it may have on the simplified 2‐D isotropic medium.

Recent work into the effect of scan coil dynamics has shown that, when moving from traditional raster scanning, the behaviour of the scan coils can be difficult to predict, ^53^ particularly over large distances. It has been assumed in this work that the beam moves instantaneously and perfectly between scan positions, which can differ from the real case where scan coil hysteresis is present. Incorporating a scan dynamics model into the diffusion model presented in this work would allow a more detailed understanding of the usage of a scan generator and beam blanker for performing subsampling.

## Supporting information

Supporting Information

## Footnotes

1 In this paper, vectors and matrices are denoted by, respectively, boldface small and boldface capital letters.

2 Simulation codes are available upon request from the authors.
