# Supplementary material for: Diffusion distribution model for damage mitigation in scanning transmission electron microscopy
Source: J Microsc. 2024 Aug 21;297(1):57–77. doi: 10.1111/jmi.13351 (PMC11629935; doi:10.1111/jmi.13351)
Supplement: Supplementary file 1 — Supporting Information [file JMI-297-57-s001.pdf]

Supplementary Information: Diffusion Distribution for Damage Mitigation in Scanning Transmission Electron Microscopy

Amirafshar Moshtaghpour* Abner Velazco-Torrejon Daniel Nicholls
Alex W. Robinson Angus I. Kirkland Nigel D. Browning

Contents

S1 Derivation of Eq. (2) **1**

S2 Derivation of Eq. (6) **3**

S3 Derivation of Eq. (8) **4**

S4 Diffusion distribution for a continuous square disc source **5**
S4.1 Derivation of Eq. (S16) 5

S5 Derivation of Eqs. (10) **and** (11) **6**

S6 Diffusion distribution as a function of distance to the activation point **7**
S6.1 Proof of Lemma S5 9
S6.2 Proof of Lemma S6 11

S7 Diffusion distribution as a function of time **11**
S7.1 Proof of Lemma S11 13
S7.2 Proof of Lemma S12 14

S8 Proof of Theorem 1 **15**

S9 Computational complexity of diffusion distribution in STEM **15**
S9.1 Numerical experiments for time complexity of diffusion distribution 17

S1 Derivation of Eq. (2)

We note that, firstly, that if $\phi(\boldsymbol{r},t)$ is a solution to the PDE in Eq. (1) with an initial condition $\phi(\boldsymbol{r},0)=\delta_{\boldsymbol{0}}$, then $\phi(\boldsymbol{r}-\boldsymbol{r}_0,t-t_0)$ will be a solution to Eq. (1) with an initial condition $\phi(\boldsymbol{r},t_0)=\delta_{\boldsymbol{r}_0}$. Therefore, we first solve Eq. (1) with an initial condition $\phi(\boldsymbol{r},0)=\delta_{\boldsymbol{0}}$. Secondly, it is evident from Eq. (1) that the diffusion distribution along the the l-th direction depends only on the diffusion

*Corresponding author. Email: amirafshar.moshtaghpour@rfi.ac.uk

distribution in the same l-th direction. Hence, the d-dimensional diffusion distribution can be decoupled into d single dimensional terms, following the form

$$\phi(\boldsymbol{r}, t) = \prod_{l=1}^{d} \phi_l(r_l, t). \tag{S1}$$

Inserting Eq. (S1) in Eq. (1),

$$\sum_{l=1}^{d} \frac{\partial \phi_l(r_l, t)}{\partial t} \prod_{j \neq l} \phi_j(r_j, t) = \sum_{l=1}^{d} D_l \frac{\partial^2 \phi_l(r_l, t)}{\partial t^2} \prod_{j \neq l} \phi_j(r_j, t),$$

or equivalently,

$$\sum_{l=1}^{d} \prod_{j \neq l} \phi_j(r_j, t) \left(\frac{\partial \phi_l(r_l, t)}{\partial t} - D_l \frac{\partial^2 \phi_l(r_l, t)}{\partial t^2} \right) = 0,$$

whose trivial solution is $\phi_l(r_l, t) = 0$ for all l, while the non-trivial solution should satisfy

$$\frac{\partial \phi_l(r_l, t)}{\partial t} = D_l \frac{\partial^2 \phi_l(r_l, t)}{\partial t^2}, \quad \forall l. \tag{S2}$$

We note that if $\phi_l(r_l, t)$ is a solution to an individual PDE in Eq. (S2), $\phi_l(a r_l, a^2 t)$ is also a solution for any $a \neq 0$. To avoid that scaling ambiguity, let $z := r_l^2/t$ and let $\psi(z) := \phi_l(r_l, t)$ be a candidate solution to Eq. (S2). Hence, by noting $\partial z / \partial r_l = 2 r_l / t$, we have

$$\frac{\partial \phi_l(r_l, t)}{\partial t} = \psi'(z) \cdot \frac{\partial z}{\partial t} = -\psi'(z) \cdot \frac{z}{t}, \tag{S3}$$

$$\frac{\partial \phi_l(r_l, t)}{\partial r_l} = \psi'(z) \cdot \frac{\partial z}{\partial r_l} = \psi'(z) \cdot \frac{2 r_l}{t},$$

$$\frac{\partial^2 \phi_l(r_l, t)}{\partial r_l^2} = \psi''(z) \cdot \frac{4 r_l^2}{t^2} + \psi'(z) \cdot \frac{2}{t}. \tag{S4}$$

Substituting Eqs. (S3) and (S4) in Eq. (S2) yields the following first order homogeneous linear differential equation:

$$\psi''(z) + \frac{z + 2D_l}{4 D_l z} \psi'(z) = 0, \quad \text{or} \quad \frac{\psi''(z)}{\psi'(z)} = -\frac{z + 2D_l}{4 D_l z}. \tag{S5}$$

The solution to Eq. (S5) is

$$\psi(z) = c_1 \int_0^z q^{-\frac{1}{2}} e^{-\frac{q}{4 D_l}} \mathrm{d}q + c_2,$$

for constants $c_1, c_2 \in \mathbb{R}$. However, this solution is still not a specific solution to Eq. (S2). Since Eq. (S2) is linear, if ϕ_l is a solution, so is its partial derivatives with respect to r_l, *i.e.*,

$$\begin{aligned} \phi_l(r_l, t) = \frac{\partial \psi(z)}{\partial r_l} &= c_1 \frac{\partial z}{\partial r_l} z^{-\frac{1}{2}} e^{-\frac{z}{4 D_l}} \\ &= \frac{2 c_1}{\sqrt{t}} \cdot \exp\left(-\frac{r_l^2}{4 D_l t} \right). \end{aligned}$$

Finally, we set $c_1 = \bar{Q}_0/4\sqrt{\pi D}$ so that $\int_{\mathbb{R}} \phi_l(r_l,t)\mathrm{d}r_l = \bar{Q}_0$. Therefore, the solution to all PDE terms in Eq. (S2) becomes

$$\phi_l(r_l,t) = \frac{\bar{Q}_0}{\sqrt{4\pi D_l t}} \cdot \exp\left(-\frac{r_l^2}{4D_l t}\right). \tag{S6}$$

By substituting Eq. (S6) in Eq. (S1) and simplification of the formula, we get

$$\phi(\boldsymbol{r},t) = \frac{Q_0}{\sqrt{|4\pi \boldsymbol{D}|t^d}} \cdot \exp\left(-\frac{\boldsymbol{r}^\top \boldsymbol{D}^{-1}\boldsymbol{r}}{4t}\right), \tag{S7}$$

where $Q_0 = \bar{Q}_0^d$. Recalling that Eq. (S7) is a solution to Eq. (1) with an initial condition $\phi(\boldsymbol{r},0) = \delta_{\boldsymbol{0}}$, the desired solution is $\phi(\boldsymbol{r}-\boldsymbol{r}_0, t-t_0)$.

S2 Derivation of Eq. (6)

Inserting Eq. (5) in Eq. (3) and setting $d=2$, we have

$$\phi(\boldsymbol{r},t) = \frac{Q_0}{4\pi\sqrt{|\boldsymbol{D}|}} \int_{t_0}^{\min(t,t_0+\tau)} \frac{1}{t-t'} \exp\Big(-\frac{(\boldsymbol{r}-\boldsymbol{r}_0)^\top \boldsymbol{D}^{-1}(\boldsymbol{r}-\boldsymbol{r}_0)}{4(t-t')}\Big)\mathrm{d}t'.$$

An application of Lemma S1 below, *i.e.*, setting $a=0$, $b=1$, and $r = \frac{(\boldsymbol{r}-\boldsymbol{r}_0)^\top \boldsymbol{D}^{-1}(\boldsymbol{r}-\boldsymbol{r}_0)}{4}$, and noting $E_1(+\infty)=0$ yields

$$\phi(\boldsymbol{r},t) = \begin{cases} \frac{Q_0}{4\pi\sqrt{|\boldsymbol{D}|}} E_1\big(\frac{(\boldsymbol{r}-\boldsymbol{r}_0)^\top \boldsymbol{D}^{-1}(\boldsymbol{r}-\boldsymbol{r}_0)}{4(t-t_0)}\big), & t_0 \le t \le t_0+\tau, \\ \frac{Q_0}{4\pi\sqrt{|\boldsymbol{D}|}} \Big(E_1\big(\frac{(\boldsymbol{r}-\boldsymbol{r}_0)^\top \boldsymbol{D}^{-1}(\boldsymbol{r}-\boldsymbol{r}_0)}{4(t-t_0)}\big) - E_1\big(\frac{(\boldsymbol{r}-\boldsymbol{r}_0)^\top \boldsymbol{D}^{-1}(\boldsymbol{r}-\boldsymbol{r}_0)}{4(t-(t_0+\tau))}\big)\Big), & t > t_0+\tau, \end{cases}$$

for $\boldsymbol{r} \neq \boldsymbol{r}_0$ and

$$\phi(\boldsymbol{r}_0,t) = \begin{cases} +\infty, & t_0 \le t \le t_0+\tau, \\ \frac{Q_0}{4\pi\sqrt{|\boldsymbol{D}|}} \ln\big(\frac{t-t_0}{t-(t_0+\tau)}\big), & t > t_0+\tau. \end{cases}$$

Lemma S1. *Let* $f(r) \coloneqq \int_{t_1}^{t_2} \frac{1}{a+b(t-t')} e^{-\frac{r}{a+b(t-t')}}\mathrm{d}t'$ *for* $r \in \mathbb{R}_{\ge 0}$ *with* $a,b,t \in \mathbb{R}_{\ge 0}$ *and* $t_1 \le t \le t_2$. *Then,* $f(0) = \frac{1}{b}\ln\big(\frac{a+b(t-t_1)}{a+b(t-t_2)}\big)$ *and, for* $r \neq 0$,

$$f(r) = \frac{1}{b}\Big(E_1\big(\frac{r}{a+b(t-t_1)}\big) - E_1\big(\frac{r}{a+b(t-t_2)}\big)\Big).$$

Moreover, if $a+b(t-t_1) \neq 0$ *and* $a+b(t-t_2) \neq 0$, $f(r)$ *is continuous on* $r \in (0,\infty)$ *and is right-continuous at* $r=0$.

Proof. For $r=0$, the integral becomes $\int_{t_1}^{t_2} \frac{1}{a+b(t-t')}\mathrm{d}t' = \frac{1}{b}\ln\big(\frac{a+b(t-t_1)}{a+b(t-t_2)}\big)$. For $r \neq 0$, by a change of variable $u = \frac{r}{a+b(t-t')}$, which gives $\mathrm{d}t' = \frac{r}{bu^2}\mathrm{d}u$, giving

$$\begin{aligned} \int_{t_1}^{t_2} \frac{1}{a+b(t-t')} e^{-\frac{r}{a+b(t-t')}}\mathrm{d}t' &= \int_{\frac{r}{a+b(t-t_1)}}^{\frac{r}{a+b(t-t_2)}} \frac{1}{bu}e^{-u}\mathrm{d}u \\ &= \int_{\frac{r}{a+b(t-t_1)}}^{+\infty} \frac{1}{bu}e^{-u}\mathrm{d}u - \int_{\frac{r}{a+b(t-t_2)}}^{+\infty} \frac{1}{bu}e^{-u}\mathrm{d}u \\ &= \frac{1}{b}\Big(E_1\big(\frac{r}{a+b(t-t_1)}\big) - E_1\big(\frac{r}{a+b(t-t_2)}\big)\Big). \end{aligned}$$

It is straightforward to show that for $a + b(t - t_1) \neq 0$ and $a + b(t - t_2) \neq 0$, $f(r)$ is continuous on $(0, \infty)$. Moreover, using the above equations in reverse order we show that $f(r)$ is right continuous at $r = 0$:

$$\begin{aligned}\lim_{r\to 0^+} \frac{1}{b}\Big(E_1\big(\frac{r}{a+b(t-t_1)}\big) - E_1\big(\frac{r}{a+b(t-t_2)}\big)\Big) &= \lim_{r\to 0^+} \int_{t_1}^{t_2} \frac{1}{a+b(t-t')} e^{-\frac{r}{a+b(t-t')}} \mathrm{d}t' \\ &= \frac{1}{b} \ln\big(\frac{a+b(t-t_1)}{a+b(t-t_2)}\big).\end{aligned}$$

□

S3 Derivation of Eq. (8)

Inserting Eq. (7) in Eq. (3) and setting $d = 2$ gives

$$\phi(\boldsymbol{r}, t) = \int_{t_0}^{\min(t, t_0+\tau)} \frac{Q_0}{4\pi^2 r_s^2 (t-t')\sqrt{|\boldsymbol{D}|}} \, I \, \mathrm{d}t', \tag{S8}$$

where

$$I := \int_{\|\boldsymbol{r}'-\boldsymbol{r}_0\|_2 \le r_s} \exp\Big(-\frac{(\boldsymbol{r}-\boldsymbol{r}')^\top \boldsymbol{D}^{-1}(\boldsymbol{r}-\boldsymbol{r}')}{4(t-t')}\Big)\mathrm{d}\boldsymbol{r}'.$$

By a change of variable $\boldsymbol{r}'' = \boldsymbol{r}' - \boldsymbol{r}_0$,

$$\begin{aligned} I :=& \int_{\|\boldsymbol{r}''\|_2 \le r_s} \exp\Big(-\frac{(\boldsymbol{r}-(\boldsymbol{r}''+\boldsymbol{r}_0))^\top \boldsymbol{D}^{-1}(\boldsymbol{r}-(\boldsymbol{r}''+\boldsymbol{r}_0))}{4(t-t')}\Big)\mathrm{d}\boldsymbol{r}'' \\ =& \exp\Big(-\frac{(\boldsymbol{r}-\boldsymbol{r}_0)^\top \boldsymbol{D}^{-1}(\boldsymbol{r}-\boldsymbol{r}_0)}{4(t-t')}\Big) \cdot I_1, \end{aligned} \tag{S9}$$

where

$$I_1 := \int_{\|\boldsymbol{r}''\|_2 \le r_s} \exp\Big(-\frac{\boldsymbol{r}''^\top \boldsymbol{D}^{-1}\boldsymbol{r}''}{4(t-t')}\Big) \exp\Big(\frac{(\boldsymbol{r}-\boldsymbol{r}_0)^\top \boldsymbol{D}^{-1}\boldsymbol{r}''}{2(t-t')}\Big)\mathrm{d}\boldsymbol{r}''. \tag{S10}$$

To compute I_1 it is necessary to convert Cartesian to Polar coordinates by defining $\boldsymbol{r}'' = [r_1'', r_2'']^\top := [u\cos\theta, u\sin\theta]^\top$ and $\boldsymbol{r} - \boldsymbol{r}_0 = [r_1 - r_{0,1}, r_2 - r_{0,2}]^\top := [v\cos\alpha, v\sin\alpha]^\top$; hence

$$\mathrm{d}\boldsymbol{r}'' = \mathrm{d}r_1''\,\mathrm{d}r_2'' = u\,\mathrm{d}u\,\mathrm{d}\theta, \tag{S11}$$

$$\boldsymbol{r}''^\top \boldsymbol{D}^{-1}\boldsymbol{r}'' = u^2(D_1^{-1}\cos^2\theta + D_2^{-1}\sin^2\theta) = \frac{u^2}{2}\big((D_1^{-1}+D_2^{-1}) + (D_1^{-1}-D_2^{-1})\cos 2\theta\big), \tag{S12}$$

$$(\boldsymbol{r}-\boldsymbol{r}_0)^\top \boldsymbol{D}^{-1}\boldsymbol{r}'' = u\,v\big(D_1^{-1}\cos\theta\cos\alpha + D_2^{-1}\sin\theta\sin\alpha\big) = u\,v\,a\cos(\theta-\beta), \tag{S13}$$

where in Eq. (S13), $a := \sqrt{D_1^{-2}\cos^2\alpha + D_2^{-2}\sin^2\alpha}$ and $\beta := \arctan\big(\frac{D_1}{D_2}\tan\alpha\big)$. Substituting Eqs. (S11), (S12), and (S13) in Eq. (S10) yields

$$I_1 = \int_0^{r_s} u\exp\Big(-\frac{(D_1^{-1}+D_2^{-1})u^2}{8(t-t')}\Big)\int_0^{2\pi} \exp\Big(\frac{\frac{1}{2}u^2(D_1^{-1}-D_2^{-1})\cos 2\theta + u\,v\,a\cos(\theta-\beta)}{2(t-t')}\Big)\mathrm{d}\theta\,\mathrm{d}u,$$

which does not simplify further. However, by setting $D_1 = D_2 = D$ the Integral I_1 reduces to

$$\begin{aligned}
I_1 =& \int_0^{r_s} u \exp\Big(-\frac{u^2}{4D(t-t')}\Big)\int_0^{2\pi}\exp\Big(\frac{u\,v\cos(\theta-\alpha)}{2D(t-t')}\Big)\mathrm{d}\theta\,\mathrm{d}u,\\
=&2\pi\int_0^{r_s} u\exp\Big(-\frac{u^2}{4D(t-t')}\Big)I_0\big(\frac{u\,v}{2D(t-t')}\big)\mathrm{d}u,
\end{aligned} \tag{S14}$$

where in the second line we use the fact that

$$\int_0^{2\pi}\exp\Big(\frac{u\,v\cos(\theta-\alpha)}{2D(t-t')}\Big)\mathrm{d}\theta =2\int_0^{\pi}\exp\Big(\frac{u\,v\cos\theta}{2D(t-t')}\Big)\mathrm{d}\theta = 2\pi I_0\big(\frac{u\,v}{2D(t-t')}\big).$$

Substituting Eq. (S14) in Eq. (S9) and combining the result with Eq. (S8) results in

$$\phi(\boldsymbol{r},t) = \int_{t_0}^{\min(t,t_0+\tau)}\frac{Q_0}{2\pi r_s^2 D(t-t')}\exp\Big(-\frac{\|\boldsymbol{r}-\boldsymbol{r}_0\|_2^2}{4D(t-t')}\Big)\int_0^{r_s} u\exp\Big(-\frac{u^2}{4D(t-t')}\Big)I_0\big(\frac{u\|\boldsymbol{r}-\boldsymbol{r}_0\|_2}{2D(t-t')}\big)\mathrm{d}u\,\mathrm{d}t'.$$

S4 Diffusion distribution for a continuous square disc source

In this section we study the diffusion distribution for a continuous square disc source with half-width r_s centered at $\boldsymbol{r} = \boldsymbol{r}_0$ and activated during time $t \in [t_0, t_0+\tau]$. In this case, the corresponding source function is $h(\boldsymbol{r},t) = Q_0 \cdot h_s(\boldsymbol{r}) \cdot h_t(t)$ with

$$h_s(\boldsymbol{r}) = \begin{cases}\frac{1}{4r_s^2}, & \text{if } \|\boldsymbol{r}-\boldsymbol{r}_0\|_\infty \le r_s,\\ 0, & \text{otherwise,}\end{cases} \qquad \text{and} \qquad h_t(t) = \begin{cases}1, & \text{if } t_0 \le t \le t_0+\tau,\\ 0, & \text{otherwise,}\end{cases} \tag{S15}$$

with Q_0 in $\mathrm{u}\cdot\mathrm{s}^{-1}$. In Eq. (S15), the ℓ_∞-norm $\|\boldsymbol{u}\|_\infty := \max_i |u_i|$, which returns the maximum absolute value entry of the input vector, is used to model the square disc source. Inserting (S15) in (3) for $d = 2$ gives (see Section S4.1 for the details)

$$\begin{aligned}
\phi(\boldsymbol{r},t) = \frac{Q_0}{16 r_s^2}\int_{t_0}^{\min(t,t_0+\tau)} &\Big(\mathrm{erf}\big(\frac{r_1-r_{0,1}+r_s}{\sqrt{4D_1(t-t')}}\big) - \mathrm{erf}\big(\frac{r_1-r_{0,1}-r_s}{\sqrt{4D_1(t-t')}}\big)\Big)\\
&\cdot\Big(\mathrm{erf}\big(\frac{r_2-r_{0,2}+r_s}{\sqrt{4D_2(t-t')}}\big) - \mathrm{erf}\big(\frac{r_2-r_{0,2}-r_s}{\sqrt{4D_2(t-t')}}\big)\Big)\,\mathrm{d}t',
\end{aligned} \tag{S16}$$

where $\mathrm{erf}(v) := \frac{2}{\sqrt{\pi}}\int_0^v \exp(-u^2)\,\mathrm{d}u$ is the error function. This special case is pertinent to imaging applications using wide (or parallel) beams as in some applications of X-ray ptychography , *e.g.*, with coherently illuminated condensed aperture [1, 2].

S4.1 Derivation of Eq. (S16)

First, we note that

$$\{\boldsymbol{r}' : \|\boldsymbol{r}'-\boldsymbol{r}_0\|_\infty \le r_s\} = \{\boldsymbol{r}' : |r_1'-r_{0,1}| \le r_s \text{ and } |r_2'-r_{0,2}| \le r_s\}.$$

Inserting Eq. (S15) in Eq. (3), setting $d = 2$, and since $(\boldsymbol{r}-\boldsymbol{r}')^\top \boldsymbol{D}^{-1}(\boldsymbol{r}-\boldsymbol{r}') = \frac{1}{D_1}(r_1-r_1')^2 + \frac{1}{D_2}(r_2-r_2')^2$, gives

$$\phi(\boldsymbol{r},t) = \frac{Q_0}{16\pi r_s^2\sqrt{|\boldsymbol{D}|}}\int_{t_0}^{\min(t,t_0+\tau)} I_1\cdot I_2\,\mathrm{d}t', \tag{S17}$$

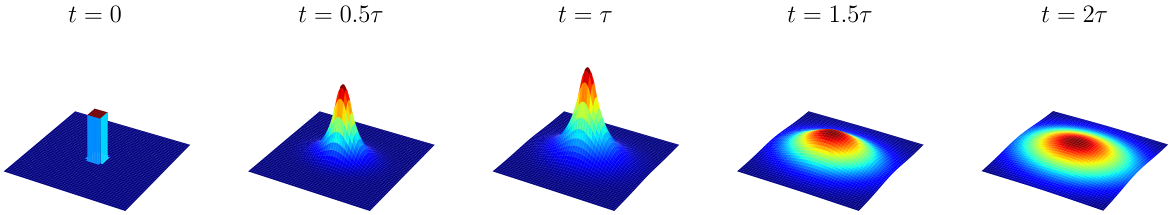

Figure S1: Diffusion distributions for a square disc source following Eq. (S16) with $\boldsymbol{D} = \mathrm{diag}(0.25, 0.5)$, $r_s = 0.2$, $\tau = 1$, $Q_0 = 1$, $t_0 = 0$, and $\boldsymbol{r}_0 = \boldsymbol{0}$. The range of vertical axes are identical.

where

$$I_i := \frac{1}{\sqrt{t-t'}} \int_{r_{0,i}-r_s}^{r_{0,i}+r_s} \exp\Big(-\frac{(r_i - r_i')^2}{4D_i(t-t')}\Big) \mathrm{d}r_i', \quad \text{for } i \in \{1,2\}.$$

An application of Lemma S2 below, *i.e.*, setting $T = 4D_i(t-t')$, $r = r_i$, $r' = r_i'$, $a_1 = r_{0,i} - r_s$, and $a_2 = r_{0,i} + r_s$, yields

$$I_i = \sqrt{\pi D_i}\Big(\mathrm{erf}\big(\frac{r_i - r_{0,i} + r_s}{\sqrt{4D_i(t-t')}}\big) - \mathrm{erf}\big(\frac{r_i - r_{0,i} - r_s}{\sqrt{4D_i(t-t')}}\big)\Big). \tag{S18}$$

Substituting Eq. (S18) in Eq. (S17) for $i \in \{1,2\}$ and noting $|\boldsymbol{D}| = D_1 D_2$ gives

$$\begin{aligned}\phi(\boldsymbol{r}, t) = \frac{Q_0}{16 r_s^2} \int_{t_0}^{\min(t, t_0+\tau)} &\Big(\mathrm{erf}\big(\frac{r_1 - r_{0,1} + r_s}{\sqrt{4D_1(t-t')}}\big) - \mathrm{erf}\big(\frac{r_1 - r_{0,1} - r_s}{\sqrt{4D_1(t-t')}}\big)\Big) \\ &\cdot\Big(\mathrm{erf}\big(\frac{r_2 - r_{0,2} + r_s}{\sqrt{4D_2(t-t')}}\big) - \mathrm{erf}\big(\frac{r_2 - r_{0,2} - r_s}{\sqrt{4D_2(t-t')}}\big)\Big)\, \mathrm{d}t'.\end{aligned}$$

Lemma S2.

$$\frac{1}{\sqrt{T}} \int_{a_1}^{a_2} e^{-\frac{(r-r')^2}{T}} \mathrm{d}r' = \frac{\sqrt{\pi}}{2}\Big(\mathrm{erf}\big(\frac{r-a_1}{\sqrt{T}}\big) - \mathrm{erf}\big(\frac{r-a_2}{\sqrt{T}}\big)\Big).$$

Proof. A change of a variable $u = \frac{(r-r')}{\sqrt{T}}$, gives $\mathrm{d}r' = -\sqrt{T}\mathrm{d}u$, giving

$$\int_{a_1}^{a_2} e^{-\frac{(r-r')^2}{T}} \mathrm{d}r' = \sqrt{T} \int_{\frac{r-a_2}{\sqrt{T}}}^{\frac{r-a_1}{\sqrt{T}}} e^{-u^2} \mathrm{d}u = \frac{\sqrt{\pi T}}{2}\Big(\mathrm{erf}\big(\frac{r-a_1}{\sqrt{T}}\big) - \mathrm{erf}\big(\frac{r-a_2}{\sqrt{T}}\big)\Big).$$

□

S5 Derivation of Eqs. (10) and (11)

Inserting Eq. (9) in Eq. (3) and setting $d = 2$ gives

$$\phi(\boldsymbol{r}, t) = Q_0 \int_{t_0}^{\min(t, t_0+\tau)} \int_{\mathbb{R}^2} f_1(\boldsymbol{r}') \cdot f_2(\boldsymbol{r}')\, \mathrm{d}\boldsymbol{r}'\, \mathrm{d}t', \tag{S19}$$

where

$$\begin{aligned} f_1(\boldsymbol{r}') &:= \frac{1}{2\pi\sqrt{|\boldsymbol{D}_s|}} \exp\Big(-\frac{1}{2}(\boldsymbol{r}' - \boldsymbol{r}_0)^\top \boldsymbol{D}_s^{-1}(\boldsymbol{r}' - \boldsymbol{r}_0)\Big), \\ f_2(\boldsymbol{r}') &:= \frac{1}{4\pi(t-t')\sqrt{|\boldsymbol{D}|}} \exp\Big(-\frac{(\boldsymbol{r}' - \boldsymbol{r})^\top \boldsymbol{D}^{-1}(\boldsymbol{r}' - \boldsymbol{r})}{4(t-t')}\Big). \end{aligned}$$

Setting $\boldsymbol{m}_1 = \boldsymbol{r}_0$, $\boldsymbol{m}_2 = \boldsymbol{r}$, $\boldsymbol{\Sigma}_1 = \boldsymbol{D}_s$, $\boldsymbol{\Sigma}_2 = 2(t-t')\boldsymbol{D}$ in Lemma S3 and solving Eq. (S19) yields

$$\phi(\boldsymbol{r},t) = \int_{t_0}^{\min(t,t_0+\tau)} \frac{Q_0}{2\pi\sqrt{|\boldsymbol{D}_e|}} \exp\big(-\frac{1}{2}(\boldsymbol{r}-\boldsymbol{r}_0)^\top \boldsymbol{D}_e^{-1}(\boldsymbol{r}-\boldsymbol{r}_0)\big)\,\mathrm{d}t', \tag{S20}$$

with $\boldsymbol{D}_e = \boldsymbol{D}_s + 2(t-t')\boldsymbol{D}$. Furthermore, assuming isotropic source and diffusion coefficients, *i.e.*, $D_{s,1} = D_{s,2} = D_s$ and $D_1 = D_2 = D$, Eq. (S20) becomes

$$\phi(\boldsymbol{r},t) = \int_{t_0}^{\min(t,t_0+\tau)} \frac{Q_0}{2\pi(D_s + 2D(t-t'))} \exp\big(-\frac{\|\boldsymbol{r}-\boldsymbol{r}_0\|_2^2}{2D_s + 4D(t-t')}\big)\,\mathrm{d}t'. \tag{S21}$$

Using Lemma S1 and setting $a = 2D_s$, $b = 4D$, and $r = \|\boldsymbol{r}-\boldsymbol{r}_0\|_2^2$ gives

$$\phi(\boldsymbol{r},t) = \begin{cases} \frac{Q_0}{4\pi D}\Big(E_1\big(\frac{\|\boldsymbol{r}-\boldsymbol{r}_0\|_2^2}{2D_s+4D(t-t_0)}\big) - E_1\big(\frac{\|\boldsymbol{r}-\boldsymbol{r}_0\|_2^2}{2D_s}\big)\Big), & t_0 \le t \le t_0+\tau, \\ \frac{Q_0}{4\pi D}\Big(E_1\big(\frac{\|\boldsymbol{r}-\boldsymbol{r}_0\|_2^2}{2D_s+4D(t-t_0)}\big) - E_1\big(\frac{\|\boldsymbol{r}-\boldsymbol{r}_0\|_2^2}{2D_s+4D(t-(t_0+\tau))}\big)\Big), & t > t_0+\tau, \end{cases}$$

and for $\boldsymbol{r} \neq \boldsymbol{r}_0$ and

$$\phi(\boldsymbol{r}_0,t) = \begin{cases} \frac{Q_0}{4\pi D}\ln\big(\frac{D_s+2D(t-t_0)}{D_s}\big), & t_0 \le t \le t_0+\tau, \\ \frac{Q_0}{4\pi D}\ln\big(\frac{D_s+2D(t-t_0)}{D_s+2D(t-t_0-\tau)}\big), & t > t_0+\tau. \end{cases}$$

Lemma S3. *Let* $\mathcal{N}(\boldsymbol{u}|\boldsymbol{m},\boldsymbol{\Sigma}) \coloneqq \frac{1}{\sqrt{|2\pi\boldsymbol{\Sigma}|}}\exp\big(-\frac{1}{2}(\boldsymbol{u}-\boldsymbol{m})^\top\boldsymbol{\Sigma}^{-1}(\boldsymbol{u}-\boldsymbol{m})\big)$*. Then*

$$\int_{\mathbb{R}^2} \mathcal{N}(\boldsymbol{u}|\boldsymbol{m}_1,\boldsymbol{\Sigma}_1)\cdot\mathcal{N}(\boldsymbol{u}|\boldsymbol{m}_2,\boldsymbol{\Sigma}_2)\,\mathrm{d}\boldsymbol{u} = \mathcal{N}(\boldsymbol{m}_1|\boldsymbol{m}_2,\boldsymbol{\Sigma}_c),$$

where $\boldsymbol{\Sigma}_c \coloneqq \boldsymbol{\Sigma}_1 + \boldsymbol{\Sigma}_2$*.*

Proof. An application of Lemma S4 and recalling that $\int_{\mathbb{R}^2}\mathcal{N}(\boldsymbol{u}|\boldsymbol{m}',\boldsymbol{\Sigma}')\,\mathrm{d}\boldsymbol{u} = 1$, for arbitrary $\boldsymbol{m}'$ and $\boldsymbol{\Sigma}'$, completes the proof. □

Lemma S4. ([3, p. 41]) *Let* $\mathcal{N}(\boldsymbol{u}|\boldsymbol{m},\boldsymbol{\Sigma}) \coloneqq \frac{1}{\sqrt{|2\pi\boldsymbol{\Sigma}|}}\exp\big(-\frac{1}{2}(\boldsymbol{u}-\boldsymbol{m})^\top\boldsymbol{\Sigma}^{-1}(\boldsymbol{u}-\boldsymbol{m})\big)$*. Then*

$$\mathcal{N}(\boldsymbol{u}|\boldsymbol{m}_1,\boldsymbol{\Sigma}_1)\cdot\mathcal{N}(\boldsymbol{u}\ \boldsymbol{m}_2,\boldsymbol{\Sigma}_2) = \mathcal{N}(\boldsymbol{m}_1|\boldsymbol{m}_2,\boldsymbol{\Sigma}_c)\cdot\mathcal{N}(\boldsymbol{u}|\boldsymbol{m}_3,\boldsymbol{\Sigma}_3),$$

where

$$\begin{aligned} \boldsymbol{\Sigma}_c &\coloneqq \boldsymbol{\Sigma}_1 + \boldsymbol{\Sigma}_2 \\ \boldsymbol{\Sigma}_3 &\coloneqq (\boldsymbol{\Sigma}_1^{-1} + \boldsymbol{\Sigma}_2^{-1})^{-1} \\ \boldsymbol{m}_3 &\coloneqq \boldsymbol{\Sigma}_3(\boldsymbol{\Sigma}_1^{-1}\boldsymbol{m}_1 + \boldsymbol{\Sigma}_2^{-1}\boldsymbol{m}_2). \end{aligned}$$

S6 Diffusion distribution as a function of distance to the activation point

Let $d_i \coloneqq \|\boldsymbol{r}-\boldsymbol{r}_i\|_2$ be the Euclidean distance from the activation location of the electron beam. We compute the first and second derivatives of ϕ_i^{on} in the following Lemma proved in Sec. S6.1.

Lemma S5. *For ϕ_i^{on} defined in Eq.* (13) *and for $d_i := \|\boldsymbol{r} - \boldsymbol{r}_i\|_2 \neq 0$ we have*

$$\frac{\partial}{\partial d_i}\phi_i^{\text{on}} = -\frac{Q_0}{2\pi D d_i}\big(e^{-\frac{d_i^2}{2D_s+4D(t-t_i)}} - e^{-\frac{d_i^2}{2D_s}}\big), \tag{S22}$$

$$\frac{\partial^2}{\partial d_i^2}\phi_i^{\text{on}} = \frac{Q_0}{2\pi D d_i^2}\Big(\big(1 + \frac{2d_i^2}{2D_s + 4D(t-t_i)}\big)e^{-\frac{d_i^2}{2D_s+4D(t-t_i)}} - \big(1 + \frac{2d_i^2}{2D_s}\big)e^{-\frac{d_i^2}{2D_s}}\Big). \tag{S23}$$

Moreover,

(i) $\frac{\partial}{\partial d_i}\phi_i^{\text{on}} < 0$, *hence, ϕ_i^{on} is a decreasing function of d_i, for all $d_i \neq 0$,*

(ii) $\frac{\partial^2}{\partial d_i^2}\phi_i^{\text{on}} < 0$, *hence, ϕ_i^{on} is a strictly concave function of d_i, if* $\frac{d_i^2}{2D_s} < \frac{1}{2}$,

(iii) $\frac{\partial^2}{\partial d_i^2}\phi_i^{\text{on}} > 0$, *hence, ϕ_i^{on} is a strictly convex function of d_i, if* $\frac{d_i^2}{2D_s+4D(t-t_i)} > \frac{1}{2}$.

Similarly, we can compute the first and second derivatives of ϕ_i^{off} using the following Lemma. See Sec. S6.2 for the proof.

Lemma S6. *For ϕ_i^{off} defined in Eq.* (14) *and for $d_i := \|\boldsymbol{r} - \boldsymbol{r}_i\|_2 \neq 0$ we have*

$$\frac{\partial}{\partial d_i}\phi_i^{\text{off}} = -\frac{Q_0}{2\pi D d_i}\big(e^{-\frac{d_i^2}{2D_s+4D(t-t_i)}} - e^{-\frac{d_i^2}{2D_s+4D(t-t_i-\tau_i)}}\big), \tag{S24}$$

$$\begin{aligned}\frac{\partial^2}{\partial d_i^2}\phi_i^{\text{off}} = \frac{Q_0}{2\pi D d_i^2}\Big(&\big(1 + \frac{2d_i^2}{2D_s + 4D(t-t_i)}\big)e^{-\frac{d_i^2}{2D_s+4D(t-t_i)}} \\ &- \big(1 + \frac{2d_i^2}{2D_s + 4D(t-t_i-\tau_i)}\big)e^{-\frac{d_i^2}{2D_s+4D(t-t_i-\tau_i)}}\Big).\end{aligned} \tag{S25}$$

Moreover,

(i) $\frac{\partial}{\partial d_i}\phi_i^{\text{off}} < 0$, *hence, ϕ_i^{off} is a decreasing function of d_i, for all $d_i \neq 0$,*

(ii) $\frac{\partial^2}{\partial d_i^2}\phi_i^{\text{off}} < 0$, *hence, ϕ_i^{off} is a strictly concave function of d_i, if* $\frac{d_i^2}{2D_s+4D(t-t_i-\tau_i)} < \frac{1}{2}$,

(iii) $\frac{\partial^2}{\partial d_i^2}\phi_i^{\text{off}} > 0$, *hence, ϕ_i^{off} is a strictly convex function of d_i, if* $\frac{d_i^2}{2D_s+4D(t-t_i)} > \frac{1}{2}$.

Lemmas S5 and S6 state that, regardless of the status of the electron beam, the diffusion distribution is always a decreasing function of the distance to the activation point, hence, the maximum value of the diffusion distribution is always at the activation point. Moreover, cases *(ii)* and *(iii)* in Lemmas S5 and S6 provide, respectively, sufficient conditions for the diffusion distribution to be a concave or convex function of distance to the activation point. These results provide an understanding of when a diffusion distribution has a global minimum or maximum, as a strictly convex (or concave) function has no more than one minimum (respectively, maximum). For example, when the condition of case *(ii)* in Lemma S6 is met, there is a point in space at a distance d_i from the activation point with the maximum number of diffusing substances compared to the other spatial points. The conditions in Lemmas S5 and S6, as illustrated in Fig. S2 (left), correspond to regions identified by a vertical line and two parabolas. As an example, from the blue region in Fig. S2(bottom), for points (d_i, t) where $2D_s(t - t_i) < d_i^2$, the diffusion distribution is a convex

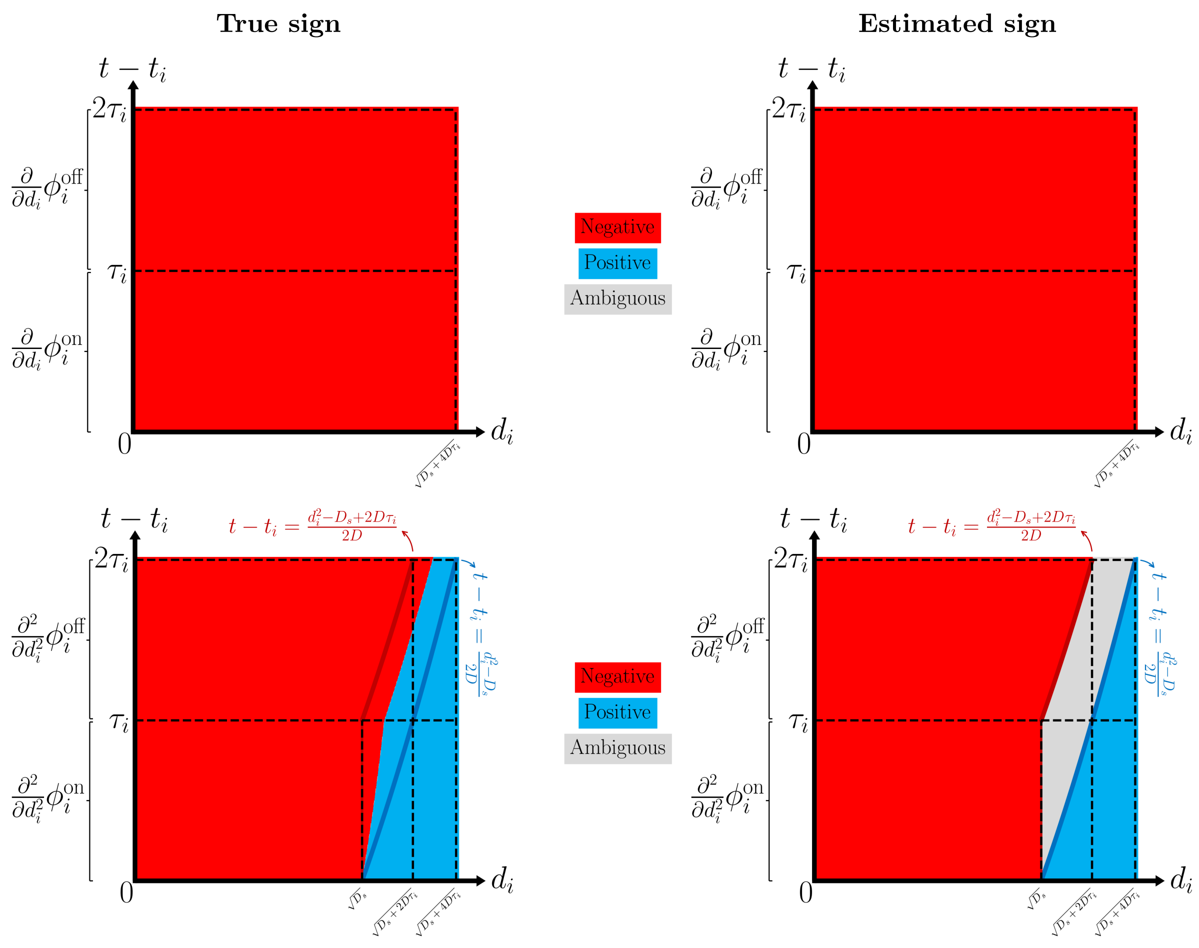

Figure S2: **Sign of the first (top) and second (bottom) derivative of the diffusion distribution from a single electron STEM probe with respect to distance from the activation point.** (Left) true sign of diffusion distribution numerically computed from Eqs. (S22), (S23), (S24), and (S25). (Right) Estimated sign following the sufficient conditions given by Cases *(ii)* and *(iii)* of Lemmas S5 and S6. The upper plots are shown for visual comparison with the plots in Fig. S3(top)

function of d_i. However, those lemmas do not cover the points where $D_s < d_i^2 < D_s + 2D(t - t_i)$, *i.e.*, an ambiguous region, as highlighted in gray in Fig. S2 (right). We note that when $D\tau_i \to 0$, the area of ambiguous region reduces to zero. Fig. S2(left) illustrates the true sign of the derivatives, numerically computed from Eqs. (S22) and (S23) for $0 < t - t_i < \tau_i$ and from Eqs. (S24) and (S25) for $\tau_i < t - t_i$.

S6.1 Proof of Lemma S5

An application of Lemma S7 by setting $v \leftarrow 0$, and $u \leftarrow d_i$, for two settings of α, *i.e.*, $\alpha \leftarrow \frac{1}{2D_s + 4D(t-t_i)}$ and $\alpha \leftarrow \frac{1}{2D_s}$, gives (S22) and (S23). Since $2D_s < 2D_s + 4D(t - t_i)$, it is evident that

$$e^{-\frac{d_i^2}{2D_s + 4D(t-t_i)}} > e^{-\frac{d_i^2}{2D_s}},$$

hence, $\frac{\partial}{\partial d_i}\phi_i^{\text{on}} < 0$ and ϕ_i^{on} is a decreasing function of d_i, for all $d_i \neq 0$.

We now prove the cases *(ii)* and *(iii)*. Since $\frac{d_i^2}{2D_s+4D(t-t_i)} < \frac{d_i^2}{2D_s}$, an application of Lemma S9 by setting $u \leftarrow \frac{d_i^2}{2D_s+4D(t-t_i)}$ and $v \leftarrow \frac{d_i^2}{2D_s}$ completes the proof.

Lemma S7. *Let $f(u) \coloneqq E_1\left(\alpha\,(v-u)^2\right)$ for $u \geq 0$ with $\alpha, v \geq 0$. The first and second derivatives of f reads, for $u \neq v$,*

$$f'(u) = \frac{2}{v-u}e^{-\alpha(v-u)^2},$$
$$f''(u) = \frac{2}{(v-u)^2}\left(1+2\alpha\,(v-u)^2\right)e^{-\alpha(v-u)^2}.$$

Moreover,

$$\begin{cases} f'(u) < 0, & \text{if } u > v, \\ f'(u) > 0, & \text{if } u < v, \end{cases} \quad \text{and} \quad f''(u) > 0,$$

Proof. We define $g(u) \coloneqq \alpha\,(v-u)^2$. Then, $g'(u) = -2\alpha(v-u)$. Using a chain rule and Lemma S8,

$$f'(u) = g'(u)\;E_1'(v)\big|_{v=g(u)} = \frac{2}{v-u}e^{-\alpha(v-u)^2},$$

for $u \neq v$ and

$$\begin{aligned} f''(u) &= \frac{\partial}{\partial u}\left(\frac{2}{v-u}\right)e^{-\alpha(v-u)^2} + \frac{2}{v-u}\frac{\partial}{\partial u}\left(e^{-\alpha(v-u)^2}\right) \\ &= \frac{2}{(v-u)^2}e^{-\alpha(v-u)^2} + \frac{2}{v-u}(2\alpha)(v-u)e^{-\alpha(v-u)^2} \\ &= \frac{2}{(v-u)^2}\left(1+2\alpha\,(v-u)^2\right)e^{-\alpha(v-u)^2}. \end{aligned}$$

We now prove the second part of the Lemma. We initially note that $\exp(-w) > 0$ for $w \in \mathbb{R}$ and

$$\begin{cases} f'(u) < 0, & \text{if } \frac{1}{v-u} < 0, \text{ or } v-u < 0, \\ f'(u) > 0, & \text{if } \frac{1}{v-u} > 0, \text{ or } v-u > 0. \end{cases}$$

Moreover, it is evident that $f''(u) > 0$ for $u \geq 0$, which completes the proof. □

Lemma S8. *Let $E_1(u) \coloneqq \int_u^{+\infty} \frac{1}{v}\exp(-v)\,\mathrm{d}v$ be the Exponential integral of order one. Then,*

$$E_1'(u) = -\frac{e^{-u}}{u}, \quad \text{for} \quad u > 0.$$

Proof. The proof is a direct application of Thm. S1 by setting $g(u) \leftarrow u$, $h(u) \leftarrow +\infty$, $t \leftarrow v$, and $f(u,t) \leftarrow \frac{1}{v}\exp(-v)$. □

Theorem S1. (**Leibniz integral rule** [4, p. 156]) *Let $g(u)$ and $h(u)$ be continuously differentiable functions of u for $u_0 < u < u_1$. Let $f(u,t)$ and $\partial f(u,t)/\partial u$ be continuous for $g(u) < t < h(u)$ and $u_0 < u < u_1$. Then, for $u_0 < u < u_1$,*

$$\frac{\partial}{\partial u}\int_{g(u)}^{h(u)} f(u,t)\,\mathrm{d}t = h'(u)f(u,h(u)) - g'(u)f(u,g(u)) + \int_{g(u)}^{h(u)} \frac{\partial}{\partial u}f(u,t)\,\mathrm{d}t.$$

Lemma S9. *Let $f(u) \coloneqq (1+2u)e^{-u}$ for $u \in \mathbb{R}$ and $g(u,v) \coloneqq f(u) - f(v)$ with $u < v$. Then,*

$$\begin{cases} g(u,v) > 0, & \text{if} \quad u > \frac{1}{2}, \\ g(u,v) < 0, & \text{if} \quad v < \frac{1}{2}, \end{cases}$$

Proof. From Lemma S10, $f(u)$ is a decreasing function of u, if $u > \frac{1}{2}$. Therefore, for $u < v$ and $u > \frac{1}{2}$, $f(u) - f(v) > 0$. Similarly, $f(v)$ is an increasing function of v, if $v < \frac{1}{2}$. Therefore, for $u < v$ and $v < \frac{1}{2}$, $f(u) - f(v) < 0$. □

Lemma S10. *Let $f(u) \coloneqq (1+2u)e^{-u}$. Then,*

$$\begin{cases} f'(u) > 0, & \text{for} \quad u < \frac{1}{2}, \\ f'(u) = 0, & \text{for} \quad u = \frac{1}{2}, \\ f'(u) < 0, & \text{for} \quad u > \frac{1}{2}. \end{cases}$$

Proof. The proof is straightforward by showing that $f'(u) = (1-2u)e^{-u}$ and noting that $e^{-u} > 0$. □

S6.2 Proof of Lemma S6

We follow the same approach to the proof of Lemma S6 in Sec. S6.1. An application of Lemma S7 by setting $v \leftarrow 0$, and $u \leftarrow d_i$, for two settings of α, *i.e.*, $\alpha \leftarrow \frac{1}{2D_s+4D(t-t_i)}$ and $\alpha \leftarrow \frac{1}{2D_s+4D(t-t_i-\tau_i)}$, gives Eqs. (S24) and (S25). Since $2D_s + 4D(t-t_i-\tau_i) < 2D_s + 4D(t-t_i)$, it is evident that

$$e^{-\frac{d_i^2}{2D_s+4D(t-t_i)}} > e^{-\frac{d_i^2}{2D_s+4D(t-t_i-\tau_i)}},$$

hence, $\frac{\partial}{\partial d_i}\phi_i^{\text{off}} < 0$ and ϕ_i^{off} is a decreasing function of d_i, for all $d_i \neq 0$.

We now prove the cases *(ii)* and *(iii)*. Since $\frac{d_i^2}{2D_s+4D(t-t_i)} < \frac{d_i^2}{2D_s+4D(t-t_i-\tau_i)}$, an application of Lemma S9 by setting $u \leftarrow \frac{d_i^2}{2D_s+D(t-t_i)}$ and $v \leftarrow \frac{d_i^2}{2D_s+4D(t-t_i-\tau_i)}$ completes the proof.

S7 Diffusion distribution as a function of time

To understand the behaviour of the diffusion distribution as a function of time, we compute the first and second derivatives of ϕ_i^{on} with respect to t in the following Lemma. See the proof in Sec. S7.1

Lemma S11. *For ϕ_i^{on} defined in Eq. (13) and for $d_i \coloneqq \|\boldsymbol{r} - \boldsymbol{r}_i\|_2 \neq 0$ we have*

$$\frac{\partial}{\partial t}\phi_i^{\text{on}} = \begin{cases} \frac{Q_0}{\pi(2D_s+4D(t-t_i))} e^{-\frac{d_i^2}{2D_s+4D(t-t_i)}}, & \text{if } d_i \neq 0, \\ \frac{Q_0}{\pi(2D_s+4D(t-t_i))}, & \text{if } d_i = 0. \end{cases} \tag{S26}$$

$$\frac{\partial^2}{\partial t^2}\phi_i^{\text{on}} = \begin{cases} \frac{-4Q_0 D}{\pi(2D_s+4D(t-t_i))^2} e^{-\frac{d_i^2}{2D_s+4D(t-t_i)}} \left(1 - \frac{d_i^2}{2D_s+4D(t-t_i)}\right), & \text{if } d_i \neq 0, \\ \frac{-4Q_0 D}{\pi(2D_s+4D(t-t_i))^2}, & \text{if } d_i = 0. \end{cases} \tag{S27}$$

Moreover,

(i) $\frac{\partial}{\partial t}\phi_i^{\mathrm{on}} > 0$*, hence,* ϕ_i^{on} *is an increasing function of* t*, for all* $t_i \le t \le \tau_i$*,*

(ii) $\frac{\partial^2}{\partial t^2}\phi_i^{\mathrm{on}} = 0$*, hence, given* d_i*,* ϕ_i^{on} *has an inflection point at time* t*, if* $\frac{d_i^2}{2D_s+4D(t-t_i)} = 1$*,*

(iii) $\frac{\partial^2}{\partial t^2}\phi_i^{\mathrm{on}} < 0$*, hence,* ϕ_i^{on} *is a strictly concave function of* t*, if* $\frac{d_i^2}{2D_s+4D(t-t_i)} < 1$*,*

(iv) $\frac{\partial^2}{\partial t^2}\phi_i^{\mathrm{on}} > 0$*, hence,* ϕ_i^{on} *is a strictly convex function of* t*, if* $\frac{d_i^2}{2D_s+4D(t-t_i)} > 1$*.*

Similarly, following Lemma, proved in Sec. S7.2, which reports the first and second derivatives of ϕ_i^{off} with respect to t.

Lemma S12. *For* ϕ_i^{off} *defined in Eq.* (14) *and for* $d_i := \|\boldsymbol{r} - \boldsymbol{r}_i\|_2 \neq 0$ *we have*

$$\frac{\partial}{\partial t}\phi_i^{\mathrm{off}} = \begin{cases} \frac{Q_0}{\pi d_i^2}\big(Ae^{-A} - Be^{-B}\big), & \text{if } d_i \neq 0, \\ \frac{-4Q_0 D\tau_i}{\pi(2D_s+4D(t-t_i))(2D_s+4D(t-t_i-\tau_i))}, & \text{if } d_i = 0. \end{cases} \tag{S28}$$

$$\frac{\partial^2}{\partial t^2}\phi_i^{\mathrm{off}} = \begin{cases} \frac{-4Q_0}{\pi d_i^4}\Big(A^2(1-A)e^{-A} - B^2(1-B)e^{-B}\Big), & \text{if } d_i \neq 0, \\ \frac{8Q_0 D^2(2D_s+4D(t-t_i-\tau_i))}{\pi(2D_s+4D(t-t_i))^2(2D_s+4D(t-t_i-\tau_i))^2}, & \text{if } d_i = 0. \end{cases} \tag{S29}$$

where

$$A := \frac{d_i^2}{2D_s + 4D(t-t_i)} \quad \text{and} \quad B := \frac{d_i^2}{2D_s + 4D(t-t_i-\tau_i)}.$$

Moreover,

(i) $\frac{\partial}{\partial t}\phi_i^{\mathrm{off}} < 0$*, hence,* ϕ_i^{off} *is a decreasing function of* t*, if* $\frac{d_i^2}{2D_s+4D(t-t_i-\tau_i)} < 1$*,*

(ii) $\frac{\partial}{\partial t}\phi_i^{\mathrm{off}} > 0$*, hence,* ϕ_i^{off} *is an increasing function of* t*, if* $\frac{d_i^2}{2D_s+4D(t-t_i)} > 1$*,*

(iii) $\frac{\partial^2}{\partial t^2}\phi_i^{\mathrm{off}} < 0$*, hence,* ϕ_i^{off} *is a strictly concave function of* t*, if* $\frac{d_i^2}{2D_s+4D(t-t_i-\tau_i)} < 2+\sqrt{2}$ *and* $\frac{d_i^2}{2D_s+4D(t-t_i)} > 2-\sqrt{2}$*,*

(iv) $\frac{\partial^2}{\partial t^2}\phi_i^{\mathrm{off}} > 0$*, hence,* ϕ_i^{off} *is a strictly convex function of* t*, if* $\frac{d_i^2}{2D_s+4D(t-t_i-\tau_i)} < 2-\sqrt{2}$ *or* $\frac{d_i^2}{2D_s+4D(t-t_i)} > 2+\sqrt{2}$*,*

Lemma S11 states that when the electron beam is on, diffusion distribution is always an increasing function of time, *i.e.*, the concentration of diffusing substances increases, and reaches a maximum value at the end of the dwell time. In contrast, when the electron beam is off depending on the ratios given in *(i)* and *(ii)* in Lemma S12, diffusion distribution can increase or decrease as a function of time. Cases *(iii)* and *(iv)* in Lemmas S11 and S12 give sufficient conditions for convexity or concavity of the diffusion distribution. These conditions, when illustrated, as in Fig. S3 (left), correspond to regions identified by parabolas. Regions in time and space that are not covered by Lemma S12, *i.e.*, ambiguous regions, are highlighted in gray in Fig. S3 (left). Fig. S3(top-right) illustrates the true sign of the first derivative and Fig. S3(bottom-right) illustrates the true sign of the second derivative, both numerically computed from Eq. (S26) for $0 < t - t_i < \tau_i$ and from (S29) for $\tau_i < t - t_i$.

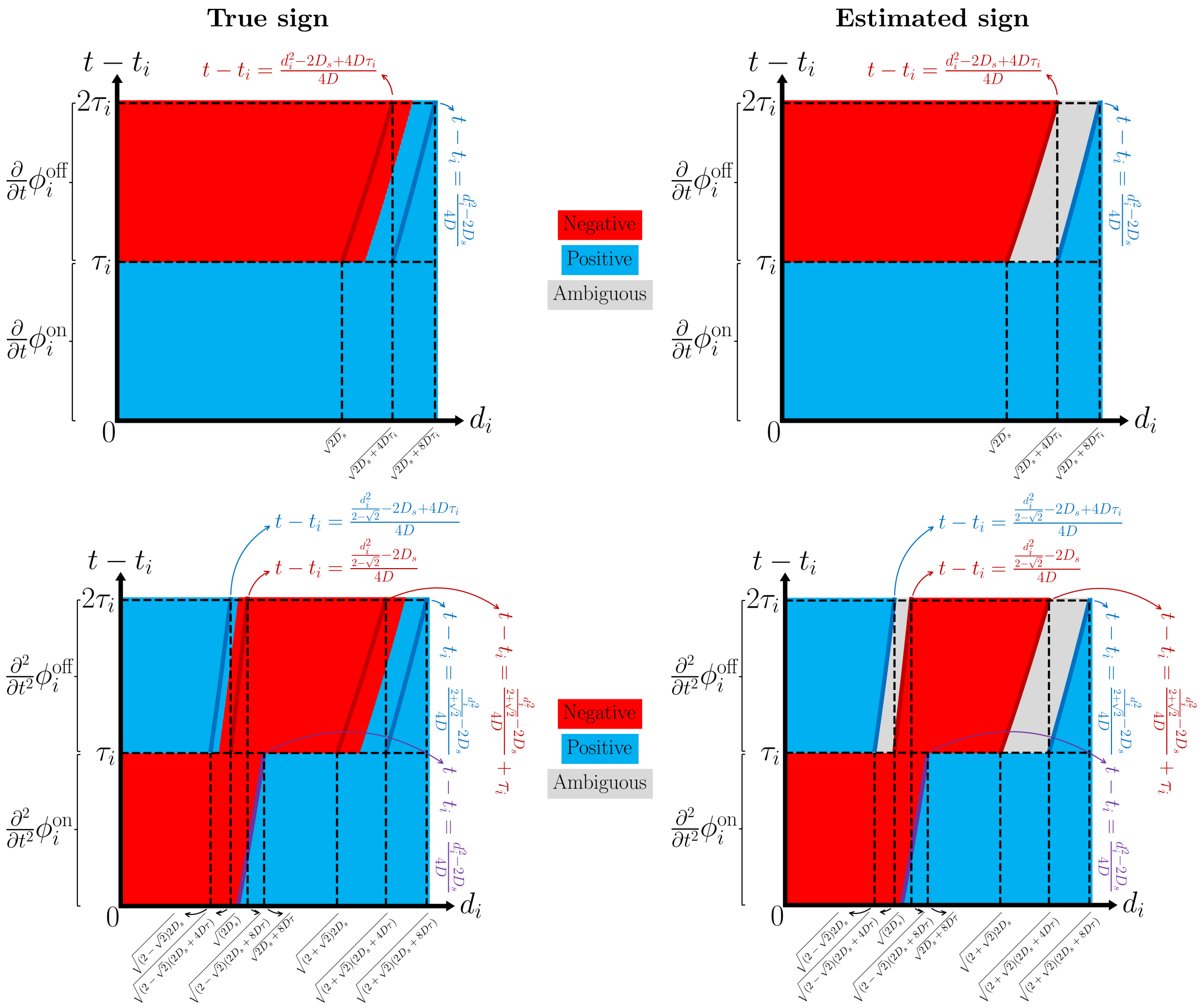

Figure S3: **Sign of the first (top) and second (bottom) derivatives of the diffusion distribution from a single STEM electron probe with respect to time.** Points with $0 < t - t_i < \tau_i$ and $\tau_i < t - t_i$ correspond to the electron beam being, respectively, on and off. True sign of the first (top-left) and second (bottom-left) derivatives are numerically computed from the equations provided by Lemmas S11 and S12. (Right) Estimated sign following the sufficient conditions given by cases *(ii)* and *(iii)* of Lemmas S11 and S12. Ambiguous regions are the pairs of $(d_i, t - t_i)$ that are not covered by the sufficient conditions in Lemmas S11 and S12.

S7.1 Proof of Lemma S11

Using Lemma S8, we first note that

$$\frac{\partial}{\partial t} E_1\left(\frac{d_i^2}{2D_s + 4D(t - t_i)}\right) = \frac{4D}{2D_s + 4D(t - t_i)} e^{-\frac{d_i^2}{2D_s + 4D(t - t_i)}} \tag{S30}$$

$$\frac{\partial}{\partial t} \ln\left(\frac{2D_s + 4D(t - t_i)}{2D_s}\right) = \frac{4D}{2D_s + 4D(t - t_i)}. \tag{S31}$$

Using Eqs. (S30) and (S31) gives

$$\frac{\partial}{\partial t}\phi_i^{\text{on}} = \begin{cases} \frac{2Q_0D_s}{2D_s+4D(t-t_i)}e^{-\frac{d_i^2}{2D_s+4D(t-t_i)}}, & \text{if } d_i \neq 0, \\ \frac{2Q_0D_s}{2D_s+4D(t-ti)}, & \text{if } d_i = 0. \end{cases} \tag{S32}$$

It is evident that $\frac{\partial}{\partial t}\phi_i^{\text{on}} > 0$. From Eq. (S32), the second derivative of ϕ_i^{on} with respect to t reads

$$\frac{\partial^2}{\partial t^2}\phi_i^{\text{on}} = \begin{cases} \frac{-8Q_0D_sD}{(2D_s+4D(t-t_i))^2}e^{-\frac{d_i^2}{2D_s+4D(t-t_i)}}\left(1-\frac{d_i^2}{2D_s+4D(t-t_i)}\right), & \text{if } d_i \neq 0, \\ \frac{-8Q_0D_sD}{(2D_s+4D(t-ti))^2}, & \text{if } d_i = 0. \end{cases} \tag{S33}$$

It is also evident that $\frac{\partial^2}{\partial t^2}\phi_i^{\text{on}} < 0$ for $\frac{d_i^2}{2D_s+4D(t-t_i)} < 1$ and $\frac{\partial^2}{\partial t^2}\phi_i^{\text{on}} > 0$ for $\frac{d_i^2}{2D_s+4D(t-t_i)} > 1$. Moreover, $\frac{\partial^2}{\partial t^2}\phi_i^{\text{on}} = 0$, if $\frac{d_i^2}{2D_s+4D(t-t_i)} = 1$.

S7.2 Proof of Lemma S12

We first note that

$$\frac{\partial}{\partial t}\ln\left(\frac{2D_s+4D(t-t_i)}{2D_s+4D(t-t_i-\tau_i)}\right) = \frac{-16D^2\tau_i}{(2D_s+4D(t-t_i))\,(2D_s+4D(t-t_i-\tau_i))}. \tag{S34}$$

Using Eqs. (S30) and (S34) gives

$$\frac{\partial}{\partial t}\phi_i^{\text{off}} = \begin{cases} \frac{2Q_0D_s}{d_i^2}\left(Ae^{-A}-Be^{-B}\right), & \text{if } d_i \neq 0, \\ \frac{-8Q_0D_sD\tau_i}{(2D_s+4D(t-t_i))(2D_s+4D(t-t_i-\tau_i))}, & \text{if } d_i = 0, \end{cases} \tag{S35}$$

where

$$A \coloneqq \frac{d_i^2}{2D_s+4D(t-t_i)} \quad \text{and} \quad B \coloneqq \frac{d_i^2}{2D_s+4D(t-t_i-\tau_i)}.$$

Since $2D_s+4D(t-t_i-\tau_i) < 2D_s+4D(t-t_i)$, then $A < B$. An application of Lemma S13 by setting $u \leftarrow A$ and $v \leftarrow B$ proves cases *(i)* and *(ii)*.

From Eq. (S35), the second derivative of ϕ_i^{off} with respect to t reads

$$\frac{\partial^2}{\partial t^2}\phi_i^{\text{on}} = \begin{cases} \frac{-8Q_0D_s}{d_i^4}\left(A^2(1-A)e^{-A}-B^2(1-B)e^{-B}\right), & \text{if } d_i \neq 0, \\ \frac{16Q_0D_sD^2(2D_s+4D(t-t_i-\tau_i))}{(2D_s+4D(t-t_i))^2(2D_s+4D(t-t_i-\tau_i))^2}, & \text{if } d_i = 0. \end{cases} \tag{S36}$$

Since $A < B$, an application of Lemma S15 by setting $u \leftarrow A$ and $v \leftarrow B$ proves the cases *(iii)* and *(iv)*.

Lemma S13. *Let $f(u) \coloneqq ue^{-u}$ for $u \in \mathbb{R}$ and $g(u,v) \coloneqq f(u) - f(v)$ with $u < v$. Then,*

$$\begin{cases} g(u,v) > 0, & \text{if} \quad u > 1, \\ g(u,v) < 0, & \text{if} \quad v < 1, \end{cases}$$

Proof. From Lemma S14, $f(u)$ is a decreasing function of u, if $u > 1$. Therefore, for $u < v$ and $u > 1$, $f(u) - f(v) > 0$. Similarly, $f(v)$ is an increasing function of v, if $v < 1$. Therefore, for $u < v$ and $v < 1$, $f(u) - f(v) < 0$. □

Lemma S14. *Let $f(u) \coloneqq ue^{-u}$. Then,*

$$\begin{cases} f'(u) > 0, & \text{for} \quad u < 1, \\ f'(u) = 0, & \text{for} \quad u = 1, \\ f'(u) < 0, & \text{for} \quad u > 1. \end{cases}$$

Proof. The proof is straightforward by showing that $f'(u) = (1-u)e^{-u}$ and noting that $e^{-u} > 0$. □

Lemma S15. *Let $f(u) \coloneqq u^2(1-u)e^{-u}$ for $u \in \mathbb{R}$ and $g(u,v) \coloneqq f(u) - f(v)$ with $u < v$. Then,*

$$\begin{cases} g(u,v) > 0, & \text{if} \quad v < 2-\sqrt{2} \text{ or } u > 2+\sqrt{2}, \\ g(u,v) < 0, & \text{if} \quad v < 2+\sqrt{2} \text{ and } u > 2-\sqrt{2}, \end{cases}$$

Proof. From Lemma S16, $f(u)$ is a decreasing function of u, if $u \in (2-\sqrt{2}, 2+\sqrt{2})$. Therefore, for $u < v$, $v < 2+\sqrt{2}$ and $u > 2-\sqrt{2}$, $f(u) - f(v) > 0$. Similarly, $f(v)$ is an increasing function of v, if $v \in (0, 2-\sqrt{2}) \cup (2+\sqrt{2}, +\infty)$. Therefore, for $u < v$, $f(u) - f(v) < 0$, if $v < 2-\sqrt{2}$ or $u > 2+\sqrt{2}$. □

Lemma S16. *Let $f(u) \coloneqq u^2(1-u)e^{-u}$. Then,*

$$\begin{cases} f'(u) > 0, & \text{for} \quad u \in (-\infty, 2-\sqrt{2}) \cup (2+\sqrt{2}, +\infty), \\ f'(u) = 0, & \text{for} \quad u \in \{0, 2+\sqrt{2}, 2+\sqrt{2}\}, \\ f'(u) < 0, & \text{for} \quad u \in (2-\sqrt{2}, 2+\sqrt{2}). \end{cases}$$

Proof. We note that $f'(u) = u(u^2 - 4u + 2)e^{-u}$ and $f'(u) = 0$ for $u \in \{0, 2-\sqrt{2}, 2+\sqrt{2}\}$. □

S8 Proof of Theorem 1

Since $\forall \boldsymbol{r}, \Lambda(\boldsymbol{r};\lambda) \geq 0$, we note that $\Lambda(\lambda) = \int \Lambda(\boldsymbol{r};\lambda)\, \mathrm{d}\boldsymbol{r} = 0$ if and only $\forall \boldsymbol{r}, \Lambda(\boldsymbol{r};\lambda) = 0$.

We first prove $\Lambda(\lambda) = 0 \implies \chi^{\max} \leq \lambda$ by contradiction. Assume that $\chi^{\max} > \lambda$, then, from Eq. 31, there exists a point $\boldsymbol{r}^*$ in space, such that $\chi(\boldsymbol{r}^*) > \lambda$. Eq. (29) implies that there also exists a point t^* and a probe position j^* such that $\psi_{j^*}(\boldsymbol{r}^*, t^*) > \lambda$. Therefore, since the function g is non-negative and $g(u) > 0$ for $u > 0$, $g(\psi_{j^*}(\boldsymbol{r}^*, t^*) - \lambda) > 0$. Moreover, since $\int_{t_1}^{t_N} p(\boldsymbol{r}, t)\, \mathrm{d}t' > 0$ for every location r, this implies that $\int_{t_1}^{t_N} p(\boldsymbol{r}^*, t)\, \mathrm{d}t' > 0$. Therefore, $\Lambda(\boldsymbol{r}^*;\lambda) > 0$; hence, $\Lambda(\lambda) > 0$.

We finally prove $\chi^{\max} \leq \lambda \implies \Lambda(\lambda) = 0$. If $\chi^{\max} \leq \lambda$, by definition in Eq. (31), for all spatial locations $\boldsymbol{r}$, we have $\chi(\boldsymbol{r}) \leq \lambda$. Therefore, from the definition in Eq. (29), for all spatial and temporal points $\boldsymbol{r}$ and t and for all electron probes j, $\psi_j(\boldsymbol{r}, t) \leq \lambda$; hence, $g(\psi_j(\boldsymbol{r}, t) - \lambda) = 0$. This implies that $\Lambda(\boldsymbol{r};\lambda) = 0$ for all $\boldsymbol{r}$.

S9 Computational complexity of diffusion distribution in STEM

In this section, we analyse the time complexity of computing (or simulating) the CDD in Eqs. (20) and (33) for full STEM and compressive STEM, respectively. Numerical supports are performed in Sec. S9.1. We emphasise that without the explicit formulation of a diffusion distribution in Eq. (20), it is necessary to use numerical solvers for the PDE in Eq. (1), for instance, as in [5], for which the time complexity is greater than computing the exponential integral in Eq. (15).

Full STEM. Let $\mathcal{R}_{\text{sim}}$ be a set of spatial points defined as the simulation grid. We assume that N_T temporal grids are defined during every period of two consecutive electron probes, *i.e.*, $t_i \leq t \leq t_{i+1}$. For a given time instance, the time complexity of every diffusion distribution $\phi_i(\boldsymbol{r}, t)$ in Eq. (19) scales as $O(|\mathcal{R}_{\text{sim}}| \cdot T_{E_1})$, where T_{E_1} is the time complexity required to compute the exponential integral function E_1 in (13). Hence, the time complexity of every CDD is $\psi_j(\boldsymbol{r}, t)$ is $O(j \cdot N_T \cdot |\mathcal{R}_{\text{sim}}| \cdot T_{E_1})$. Since $\sum_{j=1}^{N} j = N(N+1)/2$, the time complexity of diffusion in a full STEM scan, with N probe positions, is $O(N^2 \cdot N_T \cdot |\mathcal{R}_{\text{sim}}| \cdot T_{E_1})$. Since $|\mathcal{R}_{\text{sim}}|$ scales with the number of probe positions N, the time complexity of diffusion with respect to only the number of probe positions will be $O(N^3)$. Notice that the irreversible DID modeled in Eq. (23) shares the same time complexity.

Compressive STEM. Following the same steps as above for compressive STEM with M subsampled probe results in a time complexity of $O(M^2 \cdot N_T \cdot |\mathcal{R}_{\text{sim}}| \cdot T_{E_1})$. Therefore, the simulation of CDD of compressive STEM is computationally more tractable.

Methods for reducing time complexity of diffusion distribution in STEM. Although our explicit formulation of diffusion has orders of magnitude lower time complexity compared to approaches using PDE solvers, its application for large-scale, high-resolution, and accurate numerical analysis is limited. In the following, we provide two approaches to reduce computational time, albeit at the cost of reduced accuracy. Therefore in a subsequent section we describe approximations of the CDD in STEM.

***K*-Nearest Probes (*K*-NP).** We assume that when an electron probe is activated, only a subset of previous probes have contribution to the CDD and that the diffusion distributions of the other electron probe positions are negligible. From Eq. (20), let $\mathcal{N}_j$ with cardinality $|\mathcal{N}_j| = K$ be the set of K-nearest neighbor probes to the j-th electron probe position. Note that definition of a distance function identifying the nearest neighbor is arbitrary and can be based on, Euclidean, Manhattan, or Chebyshev distances [6]. Therefore, the CDD can be written as

$$\psi_j(\boldsymbol{r}, t) = \psi_j^{K-\text{NP}}(\boldsymbol{r}, t) + \epsilon_j^{K-\text{NP}}(\boldsymbol{r}, t), \tag{S37}$$

where

$$\psi_j^{K-\text{NP}}(\boldsymbol{r}, t) \coloneqq \phi_j(\boldsymbol{r}, t) + \sum_{i \in \mathcal{N}_j} \phi_i(\boldsymbol{r}, t) \tag{S38}$$

is the K-NP approximation of the CDD and $\epsilon_j^{K-\text{NP}}$ is the corresponding approximation error. Following the same steps as above, the time complexity of every $\psi_j(\boldsymbol{r}, t)$ with a K-NP approximation is $O(\min(j, K) \cdot N_T \cdot |\mathcal{R}_{\text{sim}}| \cdot T_{E_1})$. Since $\sum_{j=1}^{N} \min(j, K) = K(K+1)/2 + K(N-K)$, the full STEM scan with an K-NP approximation will have a time complexity of $O(K(N-K/2) \cdot N_T \cdot |\mathcal{R}_{\text{sim}}| \cdot T_{E_1})$ which is more scalable compared to that of $\psi_N(\boldsymbol{r}, t)$, *i.e.*, $O(N^2 \cdot N_T \cdot |\mathcal{R}_{\text{sim}}| \cdot T_{E_1})$. The number of nearest probes hence K controls the trade-off between computational time and accuracy.

Upper bound. For cases where the upper bound or the maximum value of the diffusion distribution is important, an upper bound on the diffusion distribution in Eq. (20) can be understood by considering Cor. 1. Let $\psi_j^{\text{UB}}(\boldsymbol{r}, t)$ be an upper bound to the CDD $\psi_j(\boldsymbol{r}, t)$. Using Cor. 1 and at a given time instance, the maximum value of the diffusion distribution happens at the activation

point, we replace the value of $\phi_i^{\rm off}(\boldsymbol{r},t)$ in Eq. (20) for $1 \le i \le j-1$ with its maximum value at the activation point; Hence,

$$\psi_j(\boldsymbol{r},t) \le \psi_j^{\rm UB}(\boldsymbol{r},t) \coloneqq \phi_j^{\rm on}(\boldsymbol{r},t) + \sum_{i=1}^{j-1} \phi_i^{\rm off}(\boldsymbol{r}_i,t). \tag{S39}$$

For a given time instance, the time complexity of every $\phi_i^{\rm off}(\boldsymbol{r}_i,t)$ in Eq. (S39) is $O(T_{E_1})$. By taking into account the j number of terms in Eq. (S39) and N_T the number of time instances, the time complexity of the diffusion distribution $\psi_j^{\rm UB}(\boldsymbol{r},t)$ is $O((j+|\mathcal{R}_{\rm sim}|)\cdot N_T \cdot T_{E_1})$. Therefore, the time complexity of diffusion in a full STEM scan using the upper bound $\psi_N^{\rm UB}(\boldsymbol{r},t)$ is $O(N(|\mathcal{R}_{\rm sim}| + N/2)\cdot N_T \cdot T_{E_1})$, which is lower than that of the diffusion distribution $\psi_N(\boldsymbol{r},t)$ by order $O(N)$.

S9.1 Numerical experiments for time complexity of diffusion distribution

To verify the time complexity analyses above, we have carried out several numerical tests using the same values as for the baseline STEM scan, reported in Tab. 1, for $\tau, Q_0, D, D_s, \Delta_p$. The remaining parameters are given below.

Fig. S4 shows the empirical and theoretical time complexities, with the latter given by $O(N^2 \cdot N_T \cdot |\mathcal{R}_{\rm sim}| \cdot T_{E_1})$ in Sec. S9. The elapsed times are averaged over 50 Monte-Carlo trials. In Fig. S4-left, $|\mathcal{R}_{\rm sim}|$ is set as N, *i.e.*, every scan step length is simulated with one pixel, $N_T = 1$, and we have simulated systems with $N \in \{1, 2^2, \cdots, 20^2\}$. We observe that the elapsed time is proportional to N^3. In Fig. S4-middle, we set $N = 2^2$ and $N_T = 1$ and simulate systems with $|\mathcal{R}_{\rm sim}| \in \{1, 10^2, \cdots, 100^2\}$. In this case we observe that the elapsed time is proportional to $|\mathcal{R}_{\rm sim}|$. Finally, in Fig. S4-right, we set $N = 2^2$ and $|\mathcal{R}_{\rm sim}| = N$ and simulate systems with $N_T \in \{1, 10, \cdots, 90\}$, where we observe that the elapsed time is proportional to N_T. These simulations confirm our theoretical bound $O(N^2 \cdot N_T \cdot |\mathcal{R}_{\rm sim}| \cdot T_{E_1})$.

The simulations have also been extended to two acceleration methods for simulating CDD. Initially we set $N = 20^2$, $N_T = 1$, and $|\mathcal{R}_{\rm sim}| = N$ with results averaged over 50 Monte-Carlo trials. From Sec. S9 the time complexity bounds of the $K-$NP approximation and upper bound approaches are respectively, $O(K(N-K/2)\cdot N_T \cdot |\mathcal{R}_{\rm sim}| \cdot T_{E_1})$ and $O(N(|\mathcal{R}_{\rm sim}| + N/2)\cdot N_T \cdot T_{E_1})$.

Fig. S5 shows the time complexity of the K-NP approximation with $K \in \{10j+1\}_{j=0}^{N^2/10-1}$. It is clear that the elapsed time is proportional to $K(N-K/2)$, as expected. The relative errors of this K-NP approach are plotted in Fig. S5. As a specific example approximating the CDD using only $K = 50\%$ of the previous probes resulted in approximately a 2% relative error. The elapsed time for simulating CDD in STEM using the upper bound approach is shown in Fig. S5, which is proportional to $N(|\mathcal{R}_{\rm sim}| + N/2)$.

References

[1] D. J. Batey, D. Claus, and J. M. Rodenburg, "Information multiplexing in ptychography," *Ultramicroscopy*, vol. 138, pp. 13–21, 2014.

[2] D. Claus and J. M. Rodenburg, "Diffraction-limited superresolution ptychography in the rayleigh–sommerfeld regime," *JOSA A*, vol. 36, no. 2, pp. A12–A19, 2019.

[3] K. B. Petersen, M. S. Pedersen *et al.*, "The matrix cookbook," *Technical University of Denmark*, vol. 7, no. 15, p. 510, 2008.

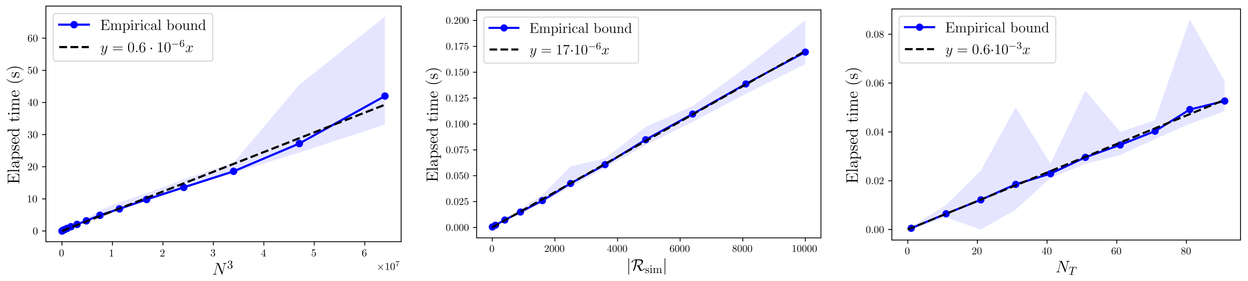

Figure S4: **Time complexity of CDD in STEM.** Number of probe positions (left); spatial resolution (middle); and temporal resolution (right).

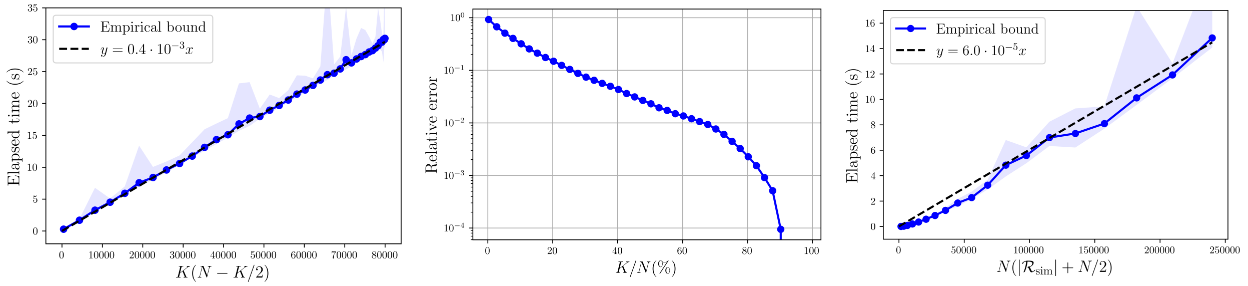

Figure S5: **Time complexity of CDD in STEM using acceleration methods.** Elapsed time for K-nearest neighbours approximation (left) with corresponding relative error (middle); Simulated elapsed time for the upper bound of CDD using (S39).

[4] J. C. Amazigo and L. A. Rubenfeld, *Advanced calculus and its applications to the engineering and physical sciences.* Wiley, 1980.

[5] D. Nicholls, J. Lee, H. Amari, A. J. Stevens, B. L. Mehdi, and N. D. Browning, "Minimising damage in high resolution scanning transmission electron microscope images of nanoscale structures and processes," *Nanoscale*, vol. 12, no. 41, pp. 21 248–21 254, 2020.

[6] É. O. Rodrigues, "Combining minkowski and chebyshev: New distance proposal and survey of distance metrics using k-nearest neighbours classifier," *Pattern Recognition Letters*, vol. 110, pp. 66–71, 2018.
